# Supplementary material for: Construction of 5-(Alkylamino)-6-aryl/alkylpyrazine-2,3-dicarbonitriles via the One-Pot Reaction of Alkyl Isocyanides with Aryl/Alkyl Carbonyl Chlorides and Diaminomaleonitrile: Fluorescence and Antimicrobial Activity Evaluation
Source: Molecules. 2022 Nov 27;27(23):8278. doi: 10.3390/molecules27238278 (PMC9736637; doi:10.3390/molecules27238278)
Supplement: Supplementary file 1 [file molecules-27-08278-s001.zip › molecules-2017817-supplementary.pdf]

Construction of 5-(Alkylamino)-6-aryl/alkylpyrazine-2,3-dicarbonitriles via the One-Pot Reaction of Alkyl Isocyanides with Aryl/Alkyl Carbonyl Chlorides and Diaminomaleonitrile: Fluorescence and Antimicrobial Activity Evaluation

Amal Al-Azmi * and Elizabeth John

Chemistry Department, Kuwait University, P.O. Box 5969, Safat 13060, Kuwait

* Correspondence: amalrchem@gmail.com or amal.alazemi@ku.edu.kw

Table of Contents

NMR Spectra **2-13**

Mass spectra **14-20**

HRMS **21-28**

Single Crystal X-ray Diffraction Studies **30-41**

NMR spectra

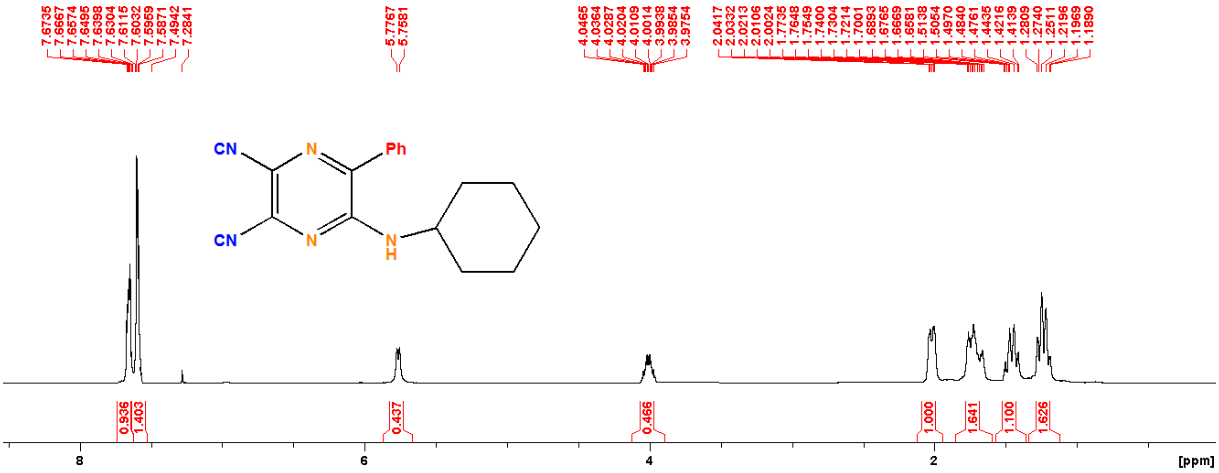

7.6735
7.6667
7.6574
7.6495
7.6398
7.6304
7.6115
7.6032
7.5959
7.5871
7.4942
7.2841
5.7767
5.7581
4.0465
4.0364
4.0287
4.0204
4.0109
4.0014
3.9938
3.9854
3.9754
2.0417
2.0332
2.0213
2.0106
2.0024
1.7735
1.7648
1.7549
1.7400
1.7304
1.7214
1.7001
1.6893
1.6765
1.6669
1.6581
1.5138
1.5054
1.4970
1.4840
1.4761
1.4435
1.4216
1.4139
1.2809
1.2740
1.2511
1.2196
1.1969
1.1890
CN
CN
N
N
Ph
N
H
0.936
1.403
0.437
0.466
1.000
1.641
1.100
1.626
8
6
4
2
[ppm]

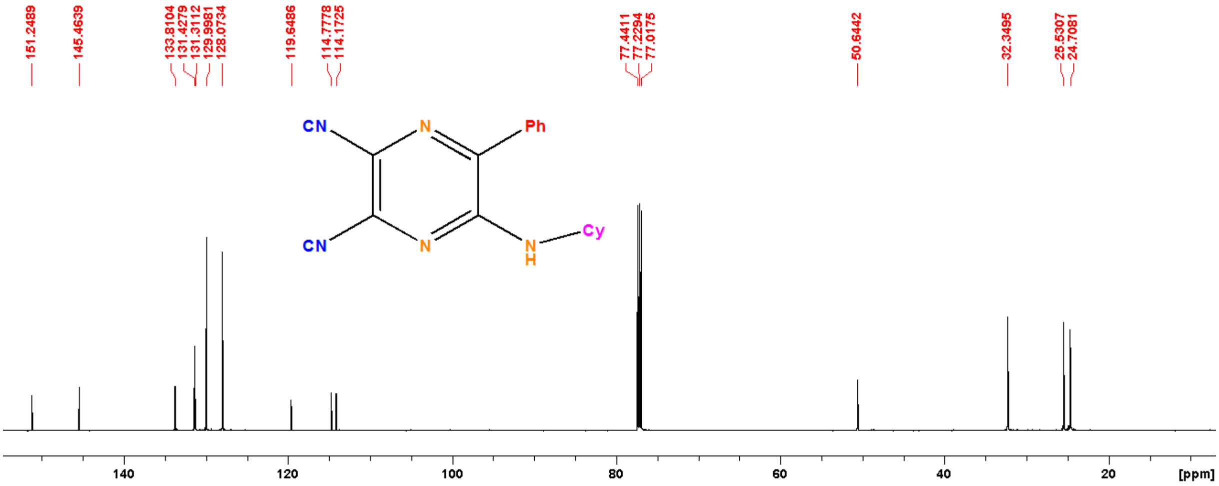

151.2489
145.4639
133.8104
131.4279
131.3112
129.9981
128.0734
119.6486
114.7778
114.1725
77.4411
77.2294
77.0175
50.6442
32.3495
25.5307
24.7081
CN
CN
N
N
Ph
N
H
Cy
140
120
100
80
60
40
20
[ppm]

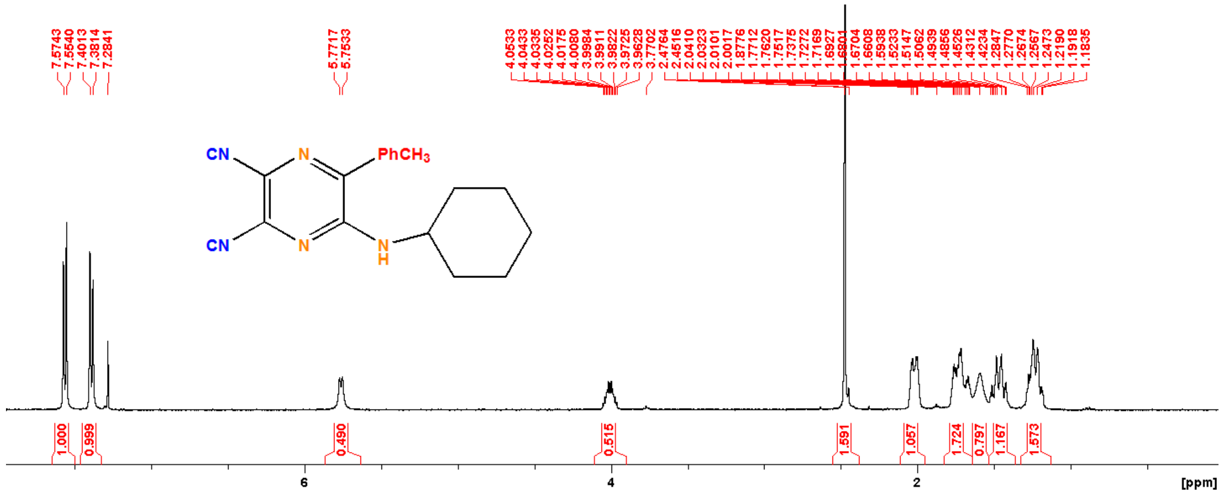
CN
CN
N
N
PhCH$_3$
N
H
6
4
2
[ppm]

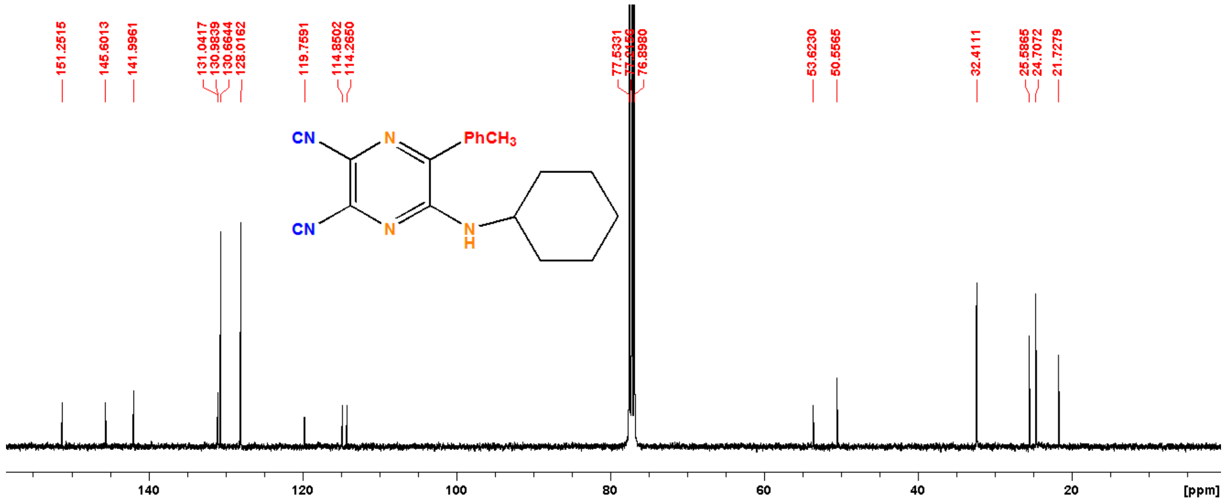
CN
CN
N
N
PhCH$_3$
N
H
140
120
100
80
60
40
20
[ppm]

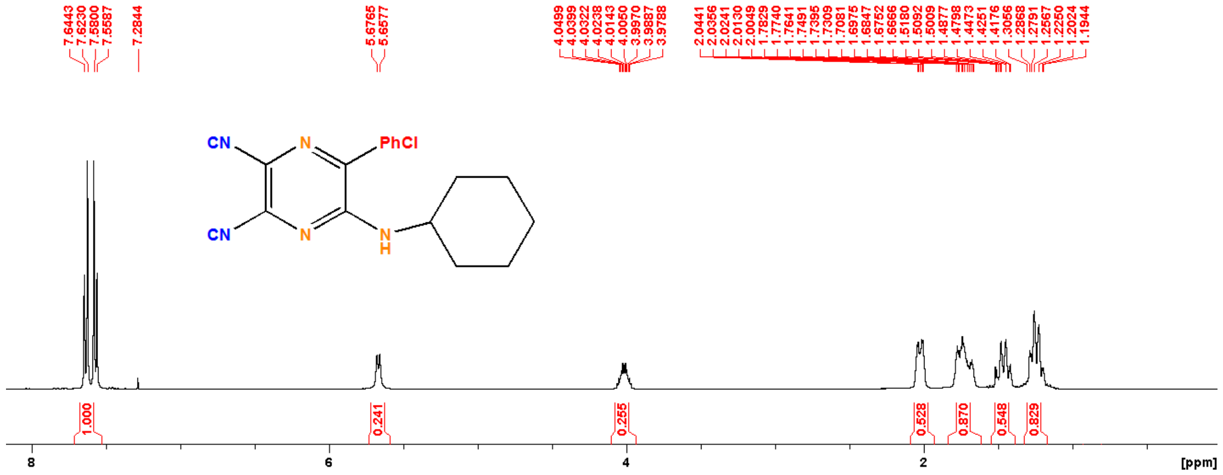
CN
CN
N
N
PhCl
N
H
8
6
4
2
[ppm]

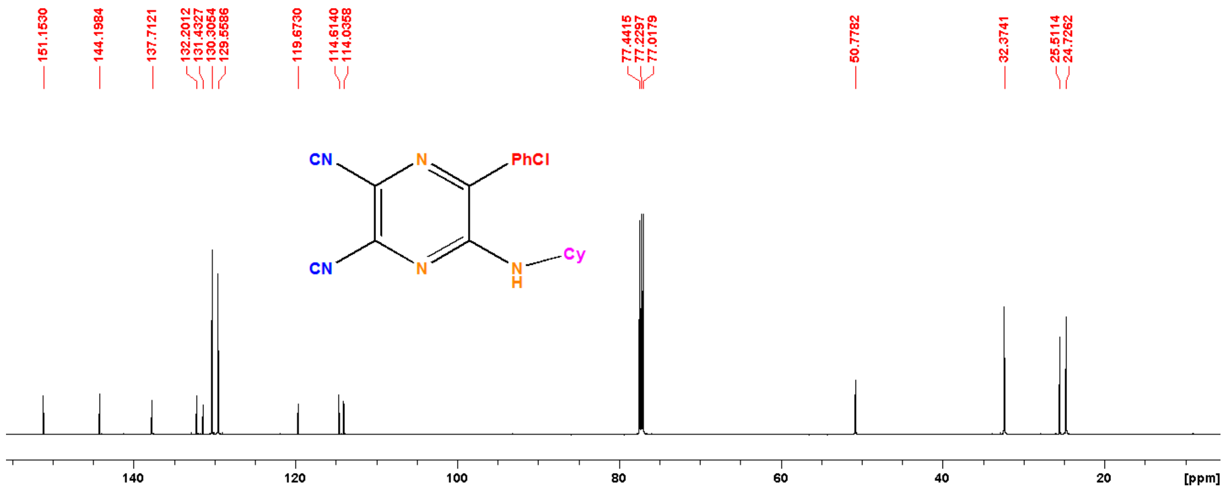
CN
N
PhCl
CN
N
N
H
Cy
[ppm]

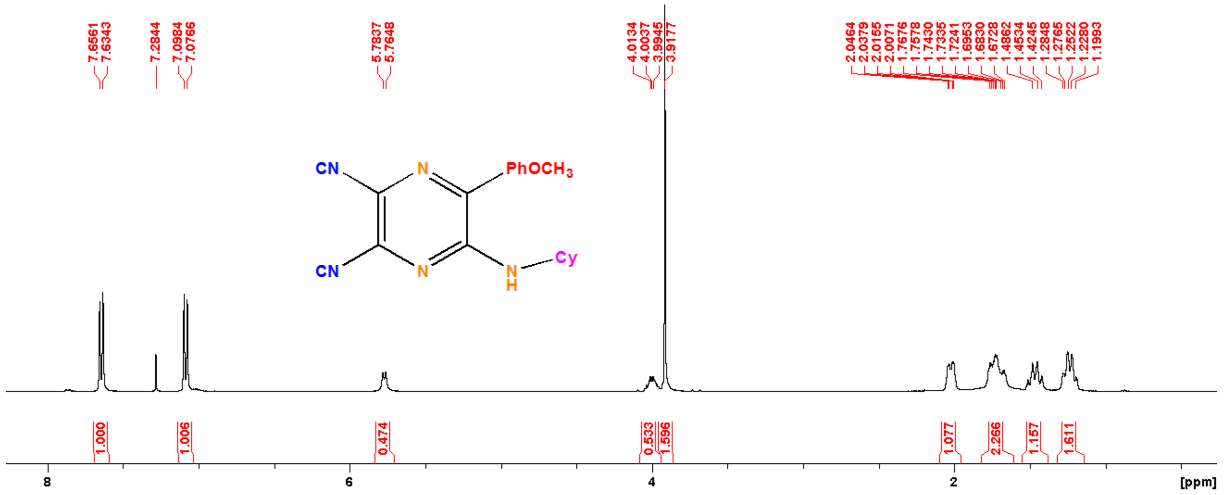
CN
N
PhOCH3
CN
N
N
H
Cy
[ppm]

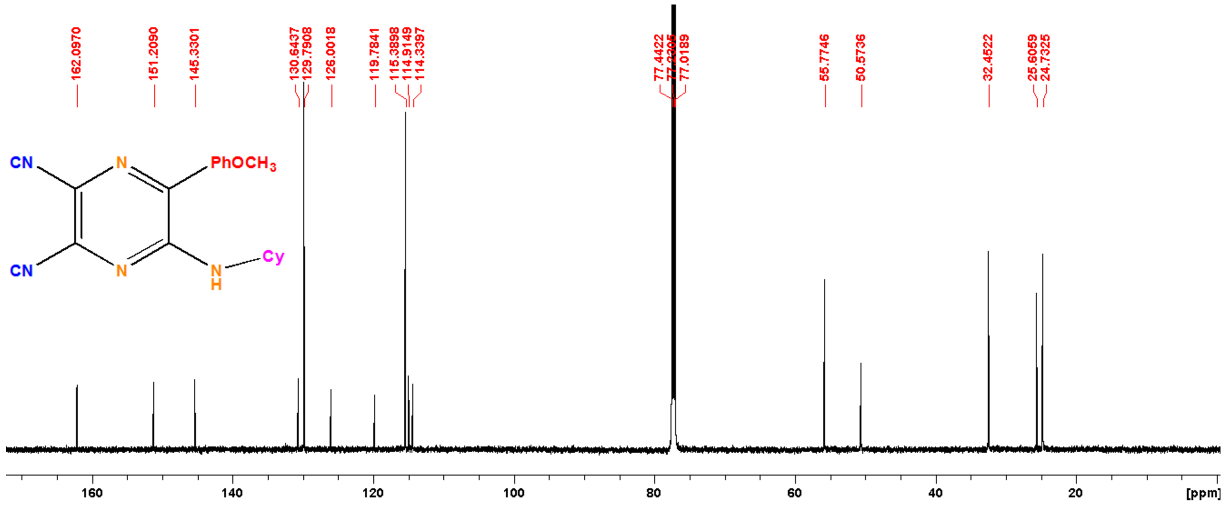
CN
N
PhOCH3
CN
N
N
H
Cy
[ppm]

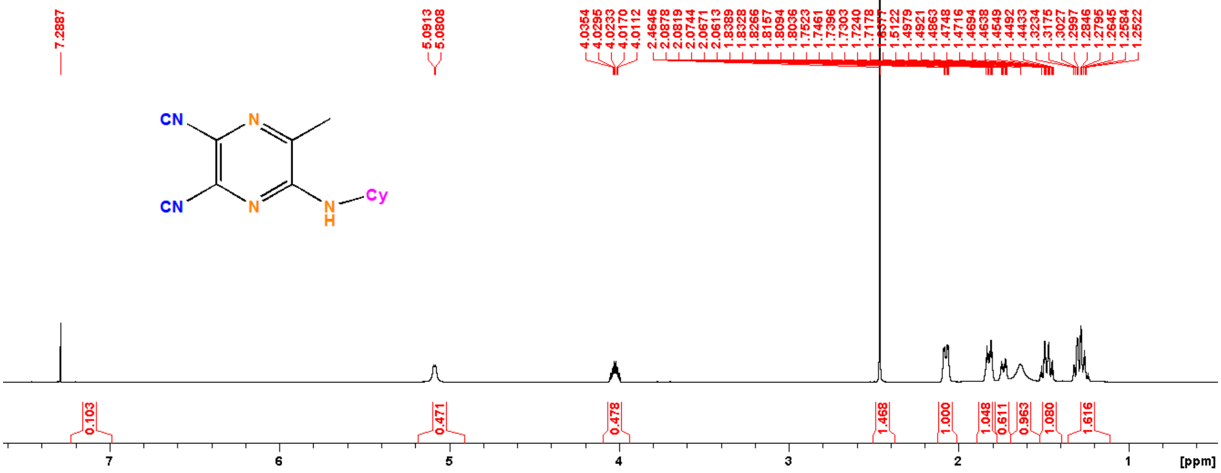
7.2887
5.0913
5.0808
CN
N
CN
N
N
H
Cy
0.103
0.471
0.478
1.468
1.000
1.048
0.963
1.080
1.616
7
6
5
4
3
2
1
[ppm]

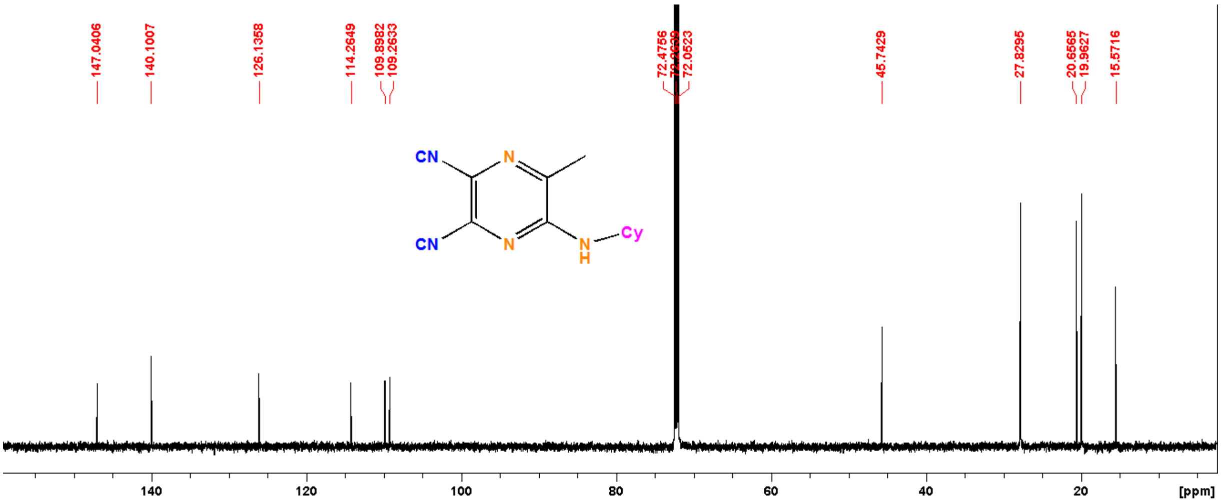
147.0406
140.1007
126.1358
114.2649
109.8982
109.2633
72.4756
72.0523
45.7429
27.8295
20.6565
19.9627
15.5716
CN
N
CN
N
N
H
Cy
140
120
100
80
60
40
20
[ppm]

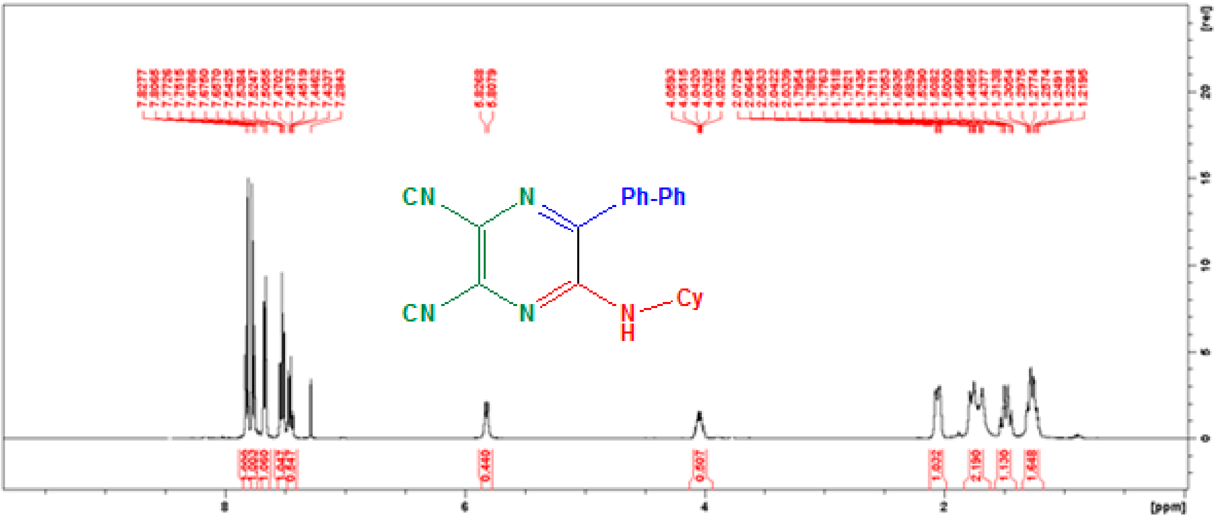
CN
N
Ph-Ph
CN
N
N
H
Cy
8
6
4
2
[ppm]

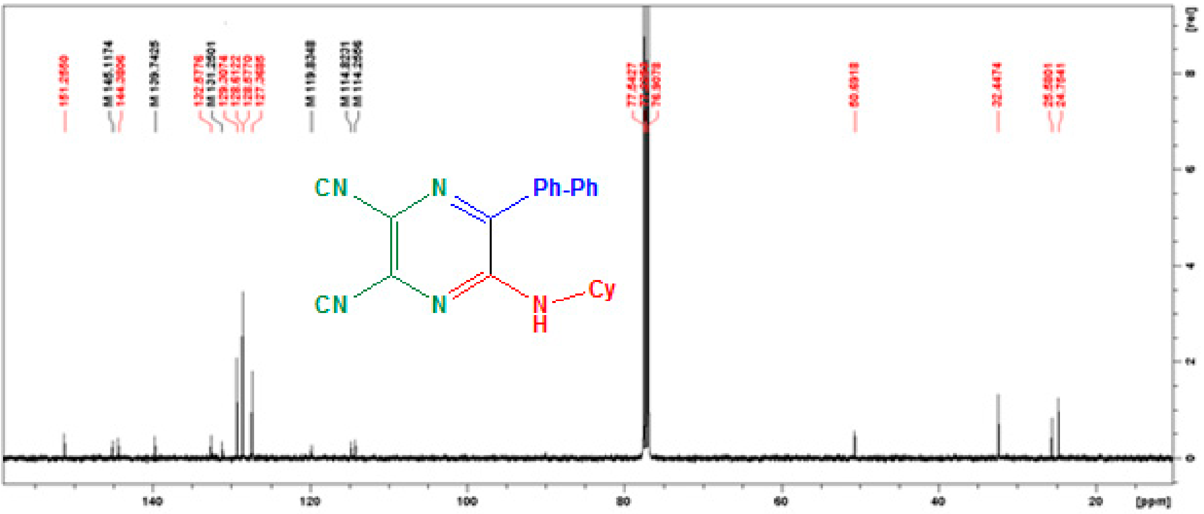
CN
N
Ph-Ph
CN
N
N
H
Cy
ppm

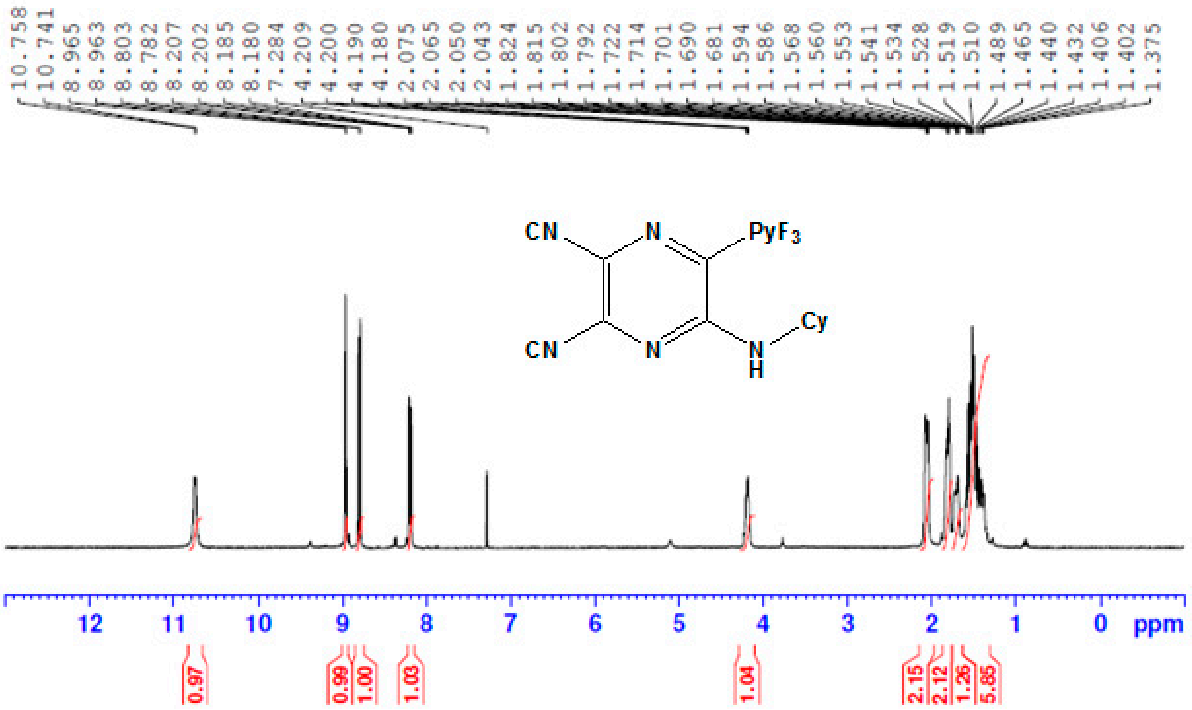
10.758
10.741
8.965
8.963
8.803
8.782
8.207
8.202
8.185
8.180
7.284
4.209
4.200
4.190
4.180
2.075
2.065
2.050
2.043
1.824
1.815
1.802
1.792
1.722
1.714
1.701
1.690
1.681
1.594
1.586
1.568
1.560
1.553
1.541
1.534
1.528
1.519
1.510
1.489
1.465
1.440
1.432
1.406
1.402
1.375
CN
N
PyF3
CN
N
N
H
Cy
ppm
0.97
0.99
1.00
1.03
1.04
2.15
2.12
1.26
5.85

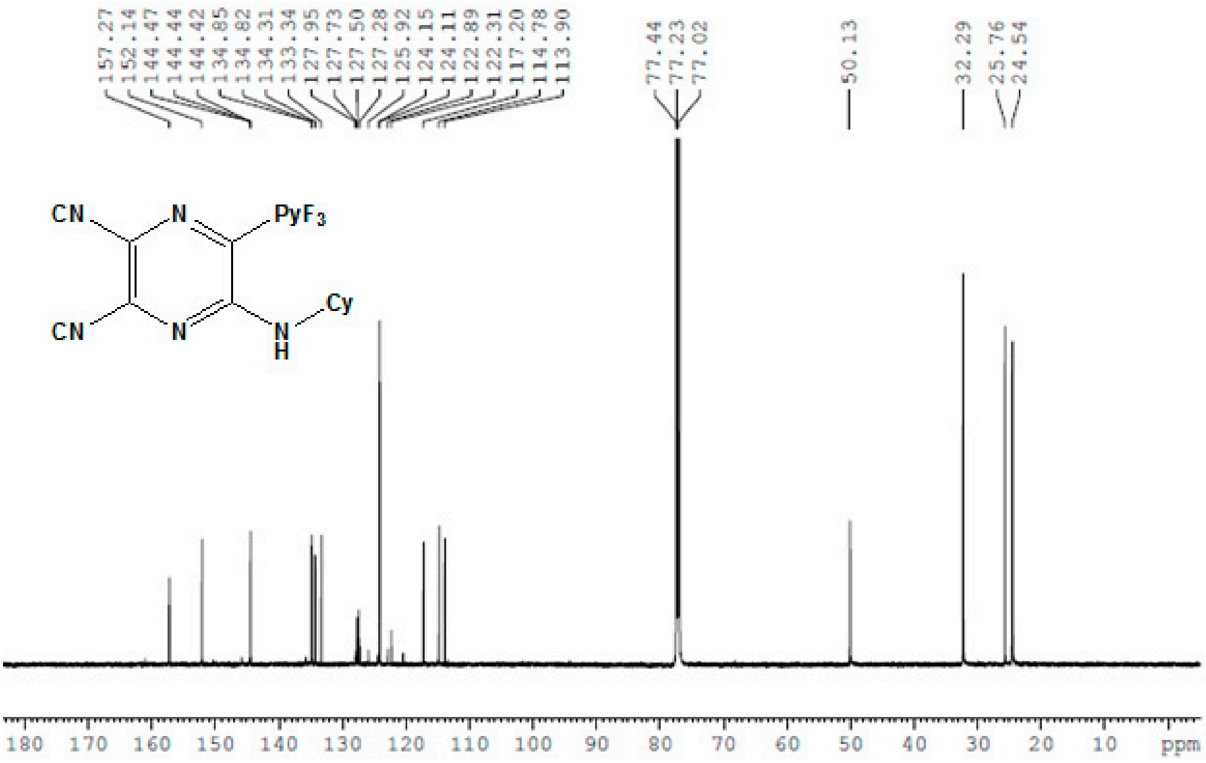

157.27
152.14
144.47
144.44
144.42
134.85
134.82
134.31
133.34
127.95
127.73
127.50
127.28
125.92
124.15
124.11
122.89
122.31
117.20
114.78
113.90
77.44
77.23
77.02
50.13
32.29
25.76
24.54
CN
CN
N
N
PyF3
Cy
N
H
180 170 160 150 140 130 120 110 100 90 80 70 60 50 40 30 20 10 ppm

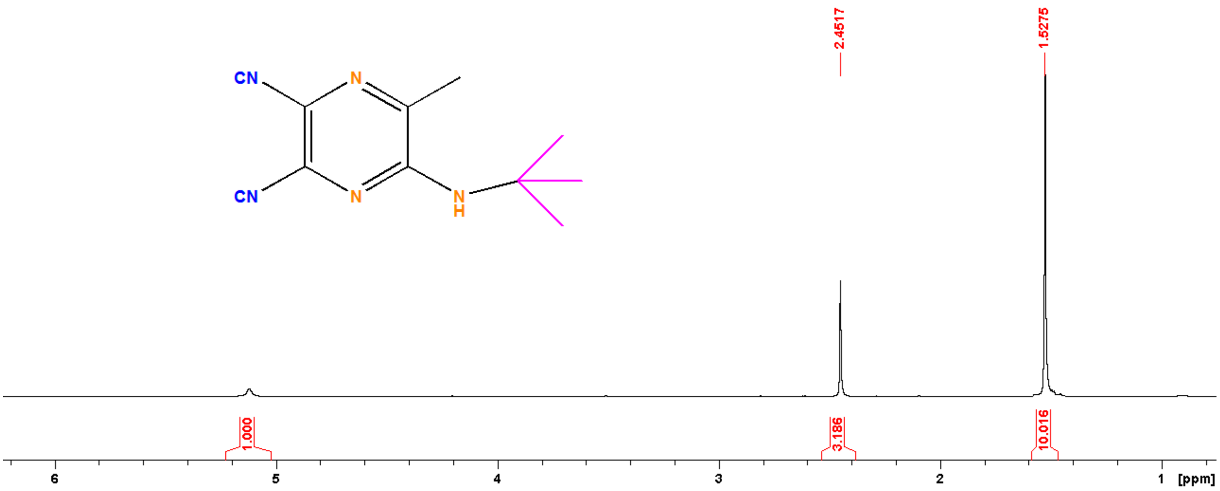

2.4517
1.5275
CN
CN
N
N
N
H
1.000
3.186
10.016
6 5 4 3 2 1 [ppm]

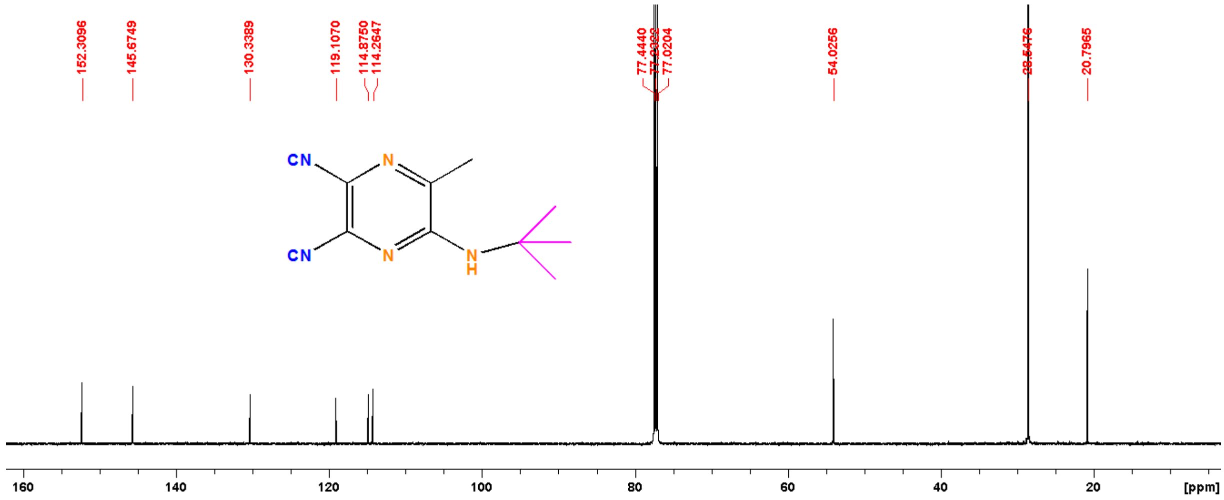
152.3096
145.6749
130.3389
119.1070
114.8750
114.2647
77.4440
77.0204
54.0256
20.7965
CN
CN
N
N
N
H
[ppm]

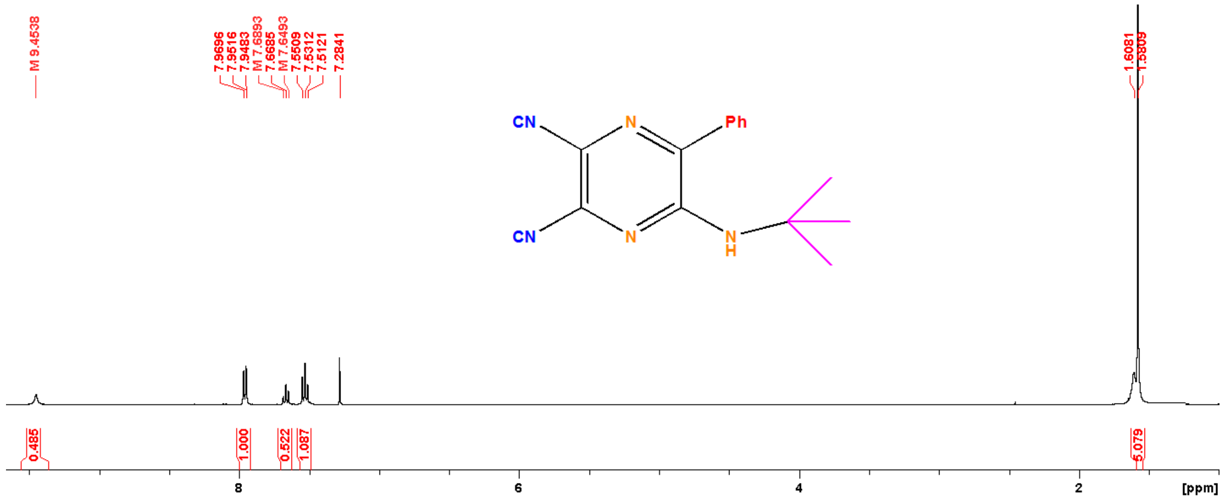
M 9.4538
7.9696
7.9516
7.9483
M 7.6893
7.6685
M 7.6493
7.5509
7.5312
7.5121
7.2841
1.6081
CN
CN
N
N
Ph
N
H
0.485
1.000
0.522
1.087
6.079
[ppm]

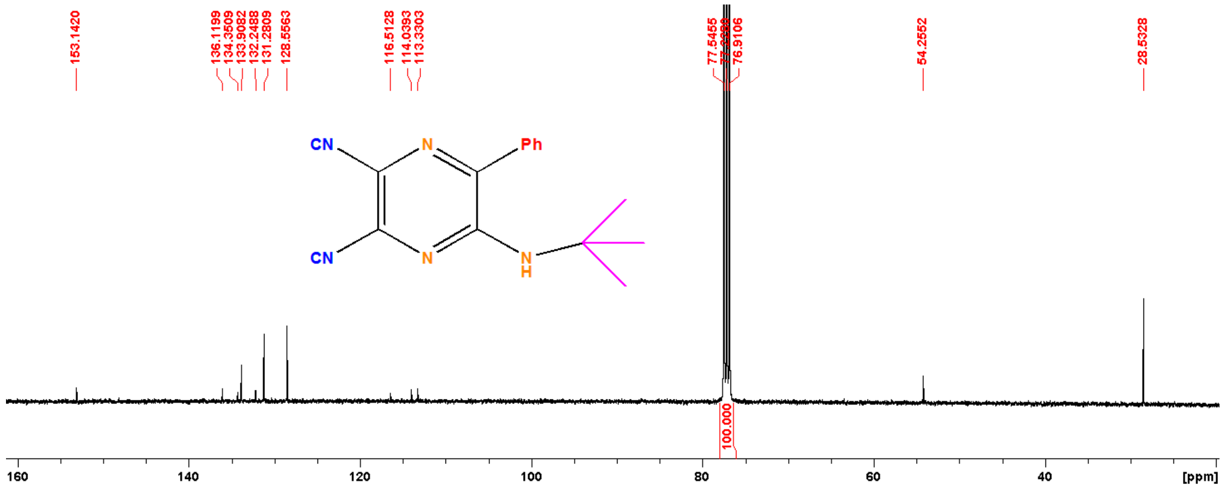
153.1420
136.1199
134.3509
133.9082
132.2488
131.2809
128.5563
116.5128
114.0393
113.3303
77.5455
76.9106
54.2552
28.5328
CN
CN
N
N
Ph
N
H
100.000
[ppm]

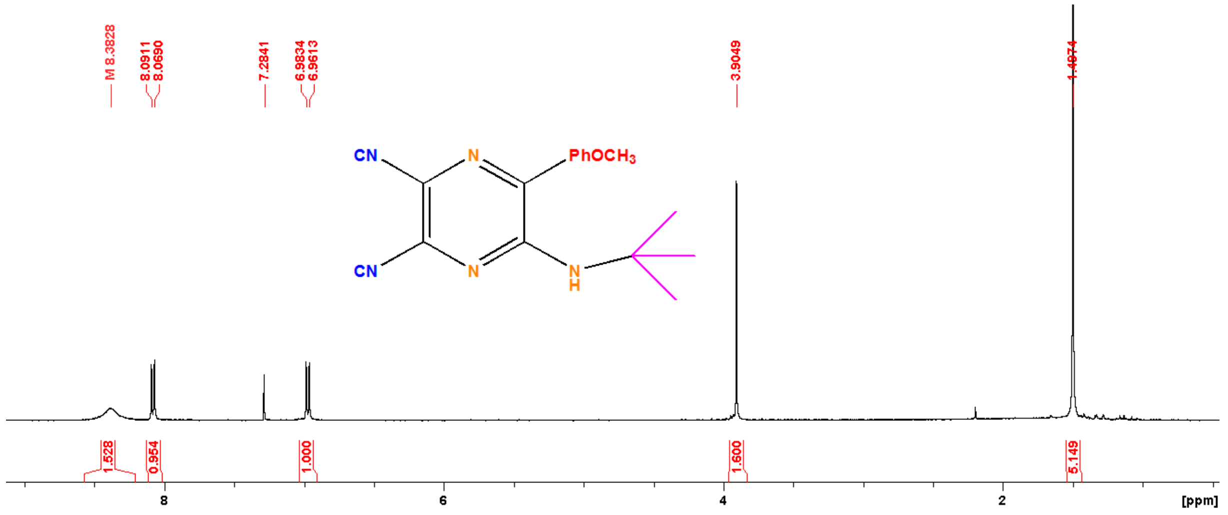
M 8.3828
8.0911
8.0690
7.2841
6.9834
6.9613
3.9049
1.4874
CN
CN
N
N
PhOCH3
N
H
1.528
0.954
1.000
1.600
5.149
8
6
4
2
[ppm]

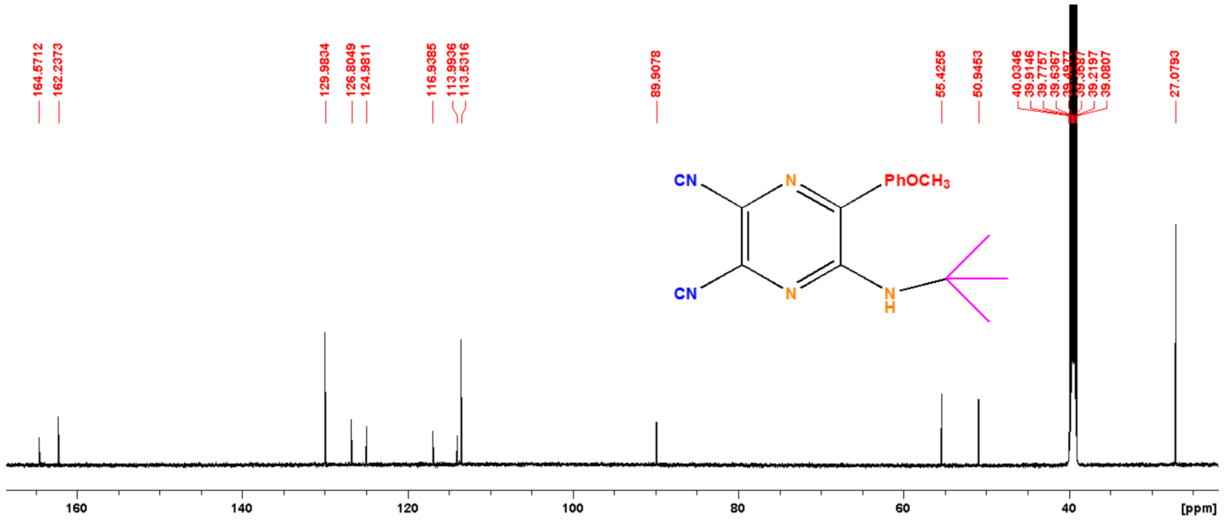
164.5712
162.2373
129.9834
126.8049
124.9811
116.9385
113.9936
113.5316
89.9078
55.4255
50.9453
40.0346
39.9146
39.7757
39.6367
39.2197
39.0807
27.0793
CN
CN
N
N
PhOCH3
N
H
160
140
120
100
80
60
40
[ppm]

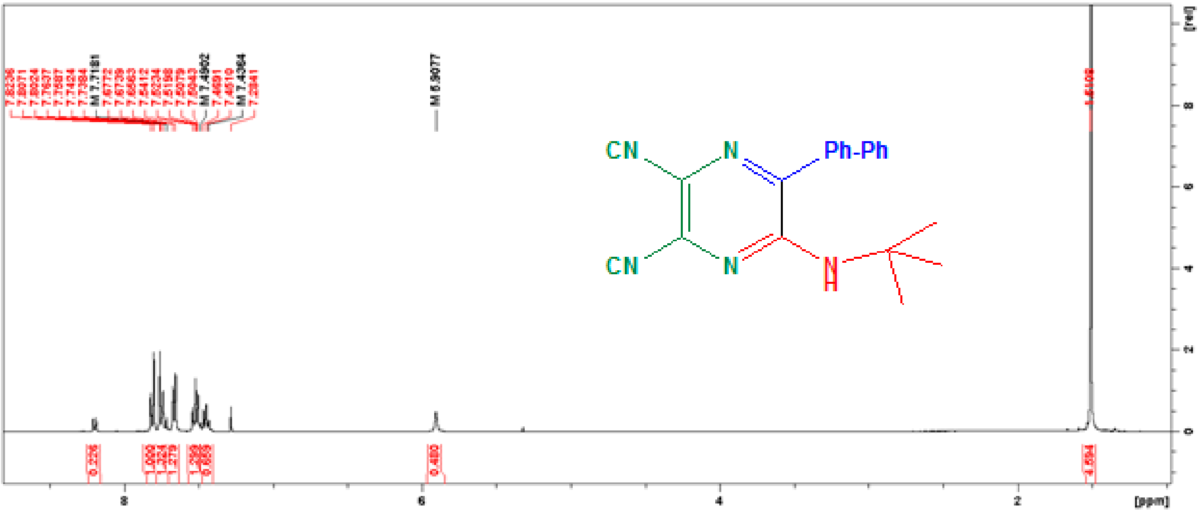

CN
CN
N
N
Ph-Ph
N
H
[ppm]
[rel]

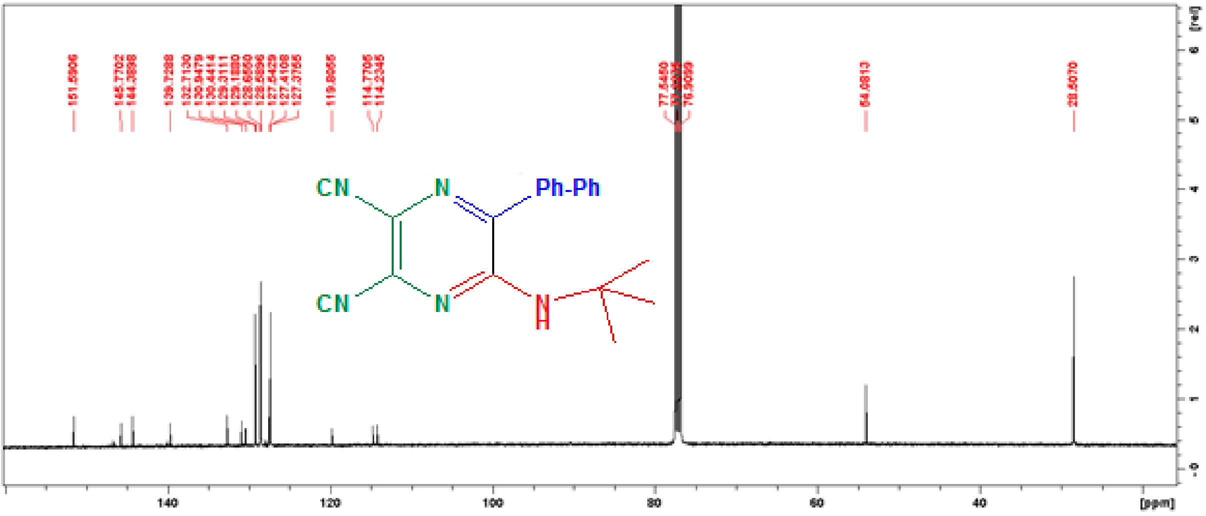

CN
CN
N
N
Ph-Ph
N
H
[ppm]
[rel]

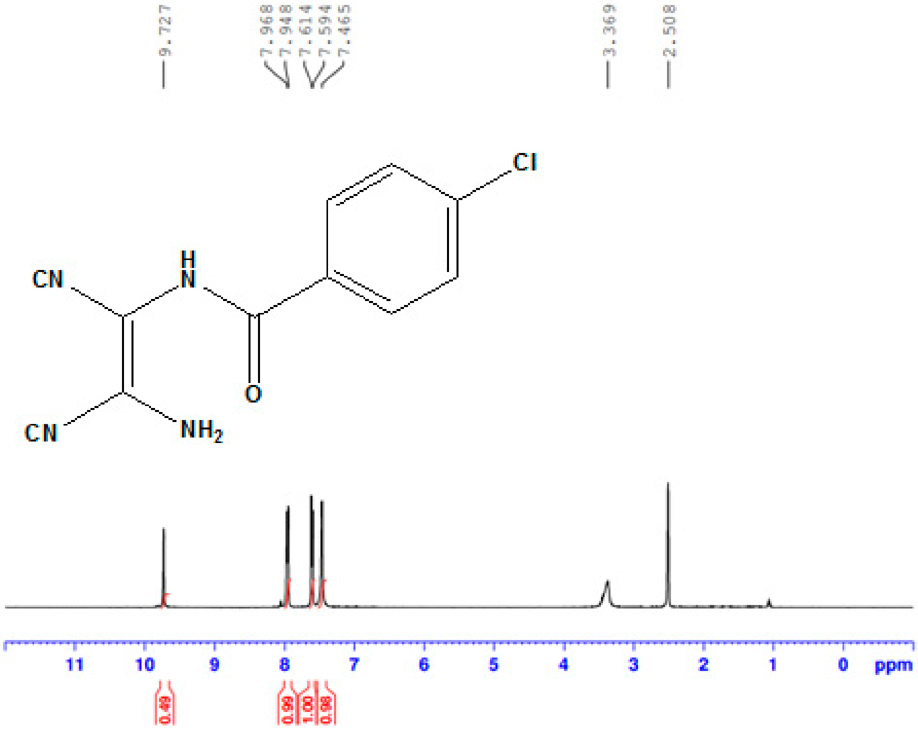
9.727
7.968
7.948
7.614
7.594
7.465
3.369
2.508
Cl
CN
H
N
O
CN
NH2
11
10
9
8
7
6
5
4
3
2
1
0
ppm
0.49
0.99
1.00
0.98

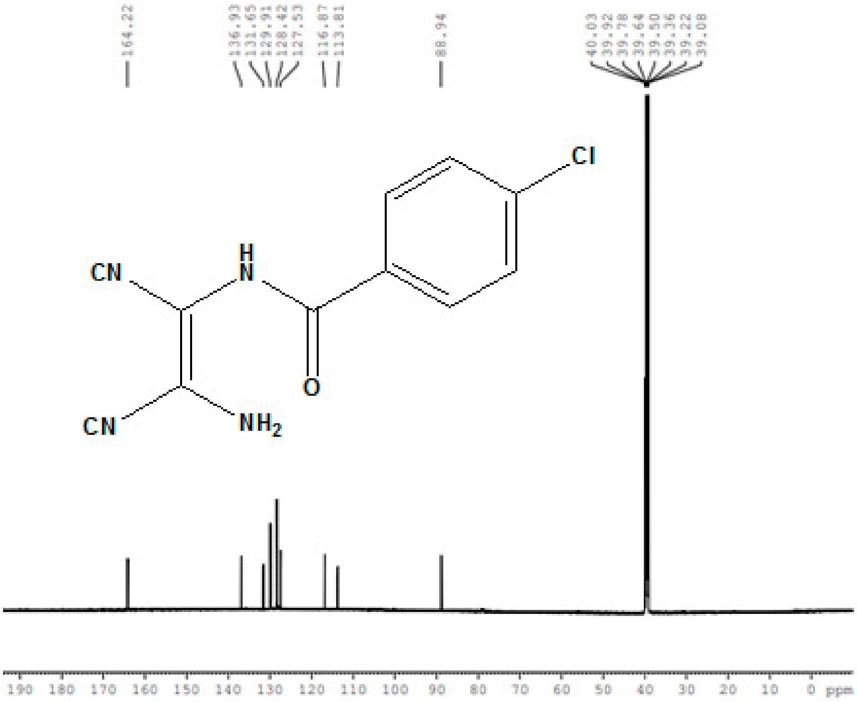
164.22
136.93
131.65
129.91
128.42
127.53
116.87
113.81
88.94
40.03
39.92
39.78
39.64
39.50
39.36
39.22
39.08
Cl
CN
H
N
O
CN
NH2
190
180
170
160
150
140
130
120
110
100
90
80
70
60
50
40
30
20
10
0
ppm

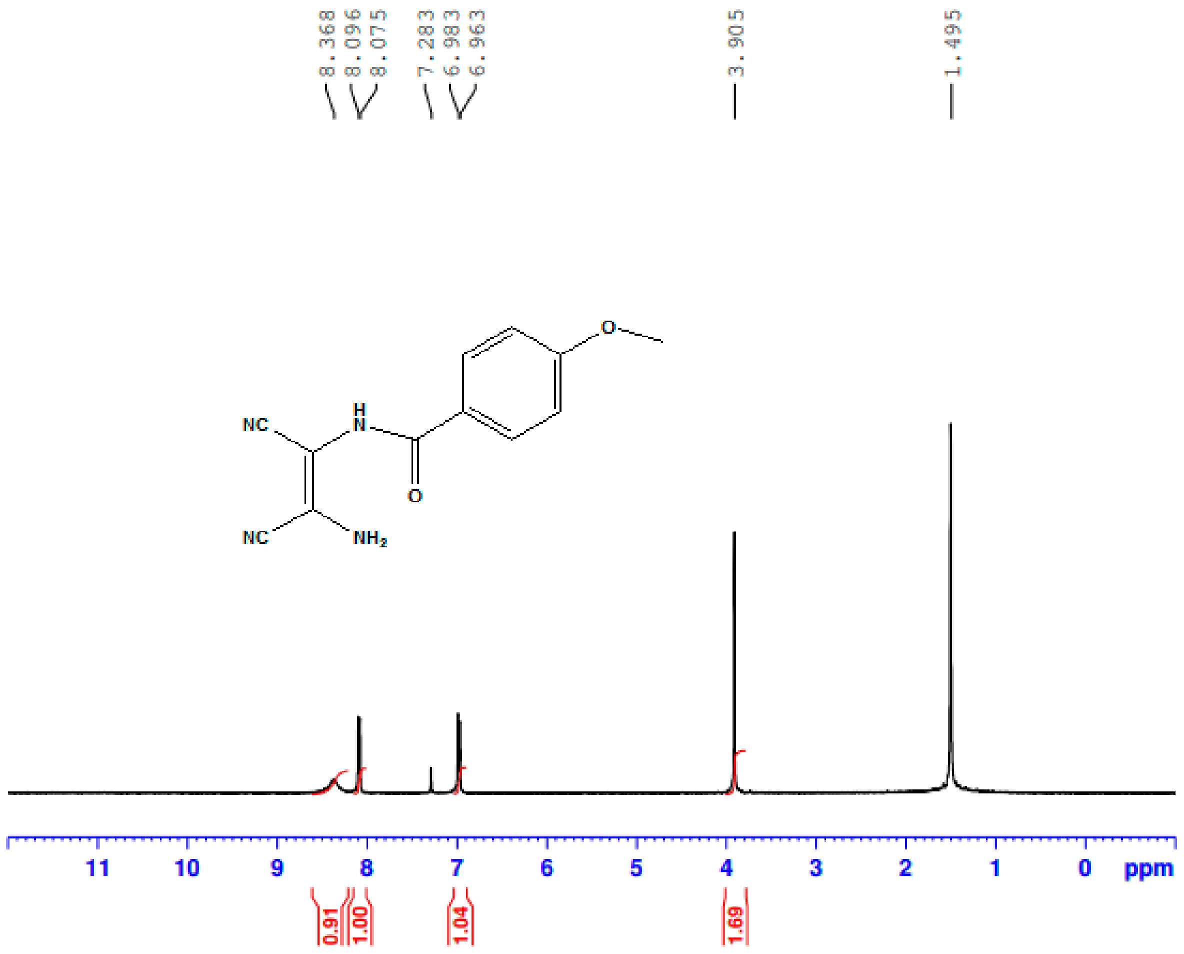
8.368
8.096
8.075
7.283
6.983
6.963
3.905
1.495
NC
H
N
O
O
NC
NH2
11
10
9
8
7
6
5
4
3
2
1
0
ppm
0.91
1.00
1.04
1.69

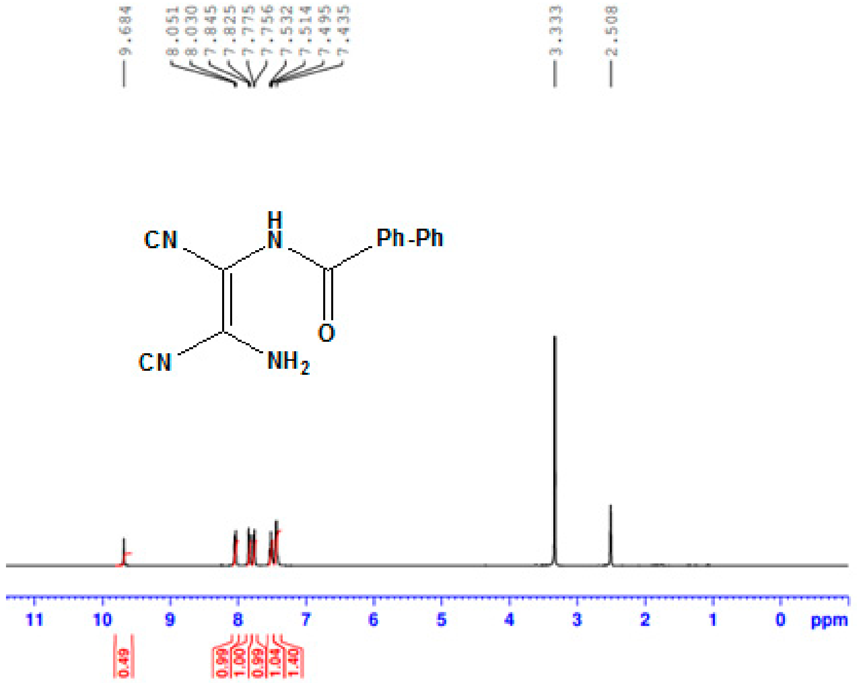

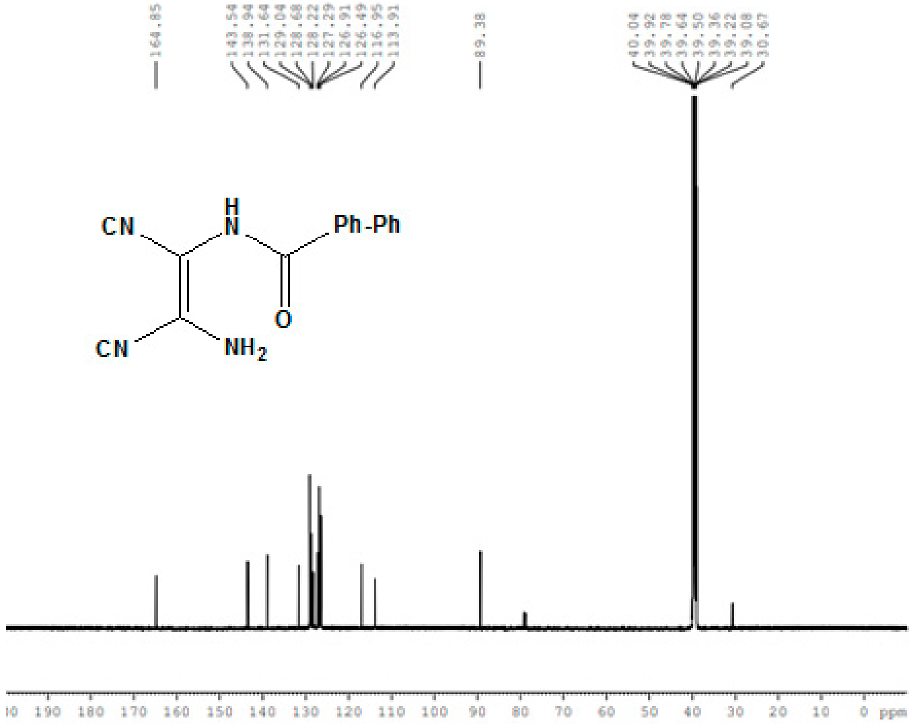

Mass spectra

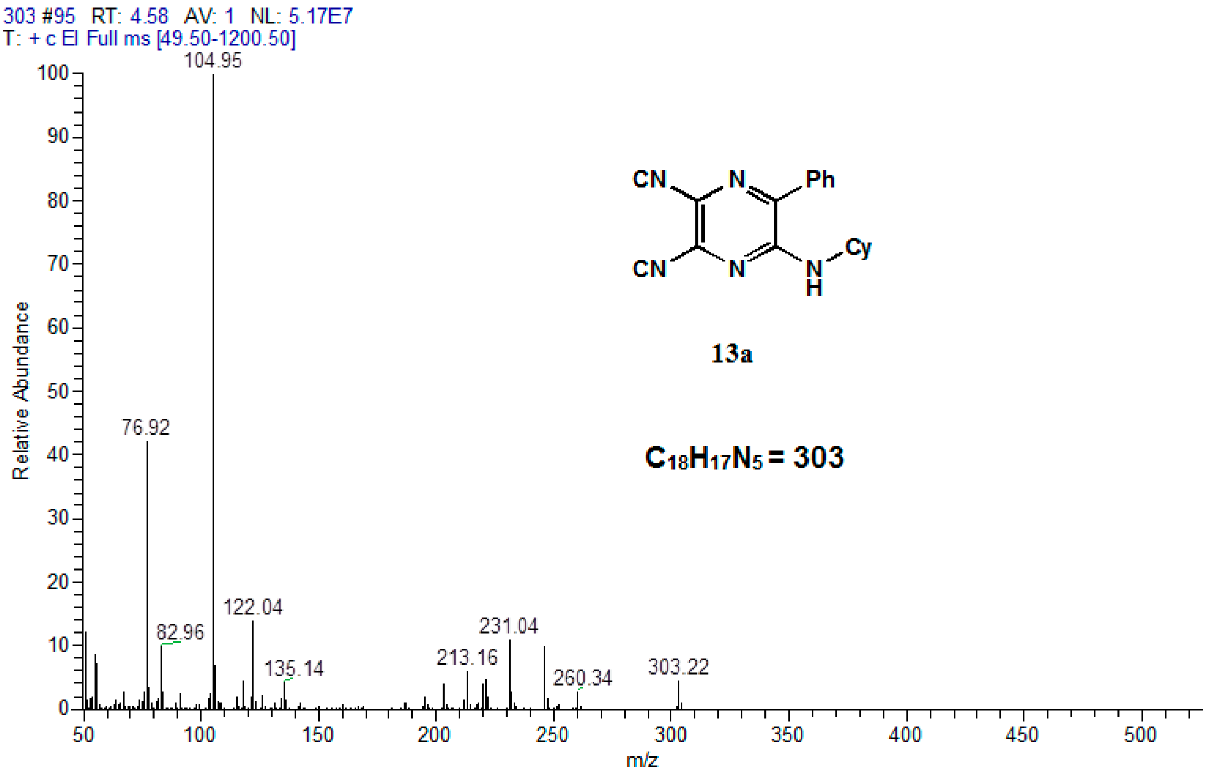

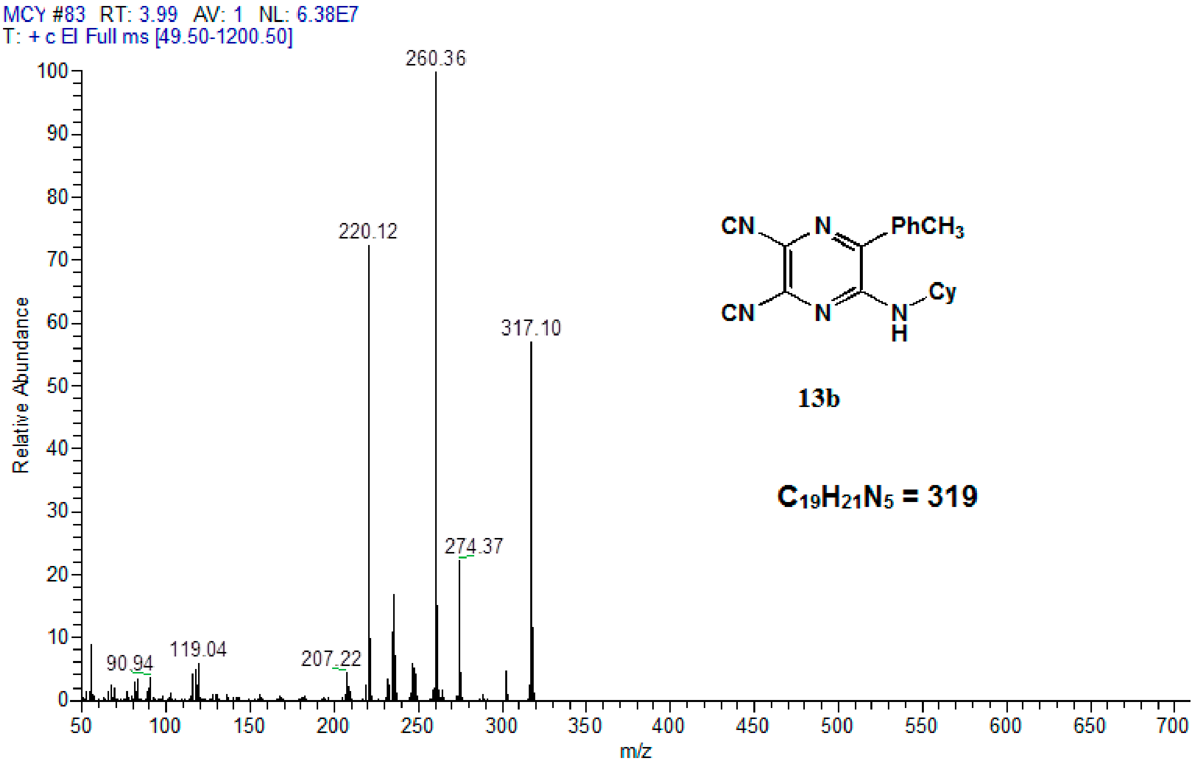

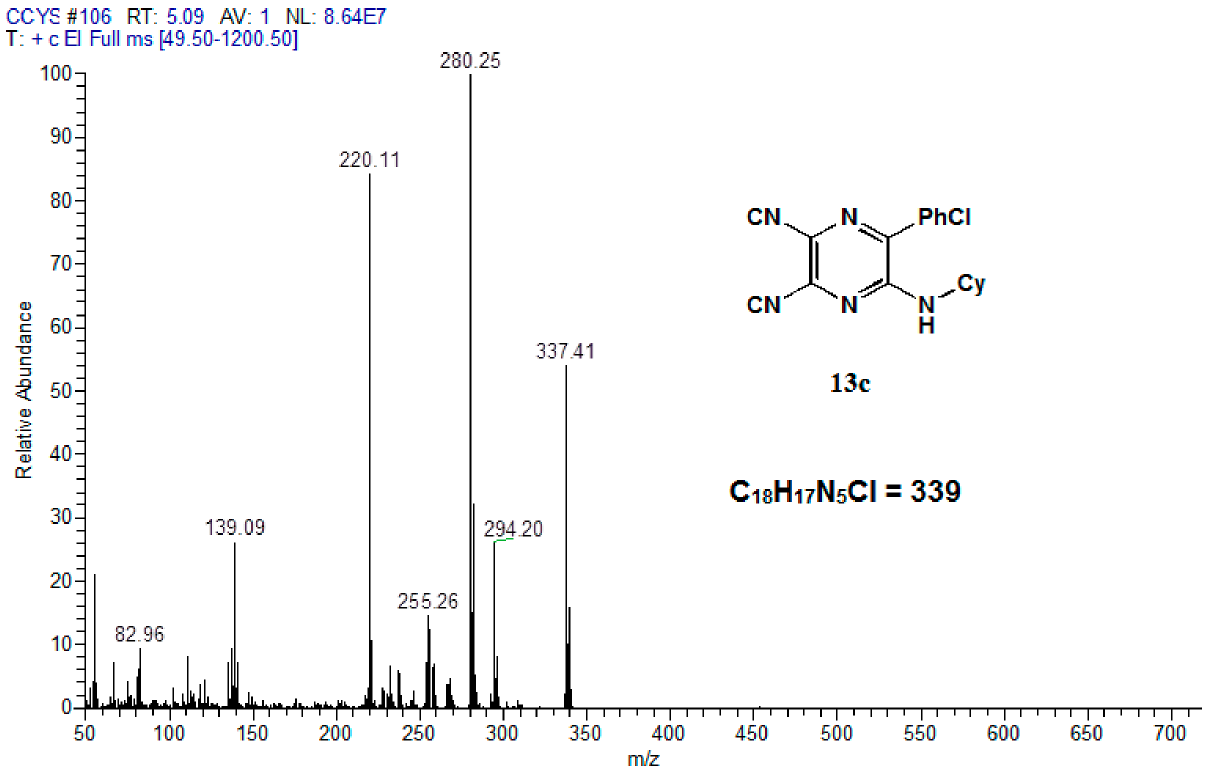
CCYS #106 RT: 5.09 AV: 1 NL: 8.64E7
T: + c EI Full ms [49.50-1200.50]
280.25
220.11
337.41
139.09
294.20
255.26
82.96
Relative Abundance
m/z
CN
N
PhCl
CN
N
N
H
Cy
13c
C18H17N5Cl = 339

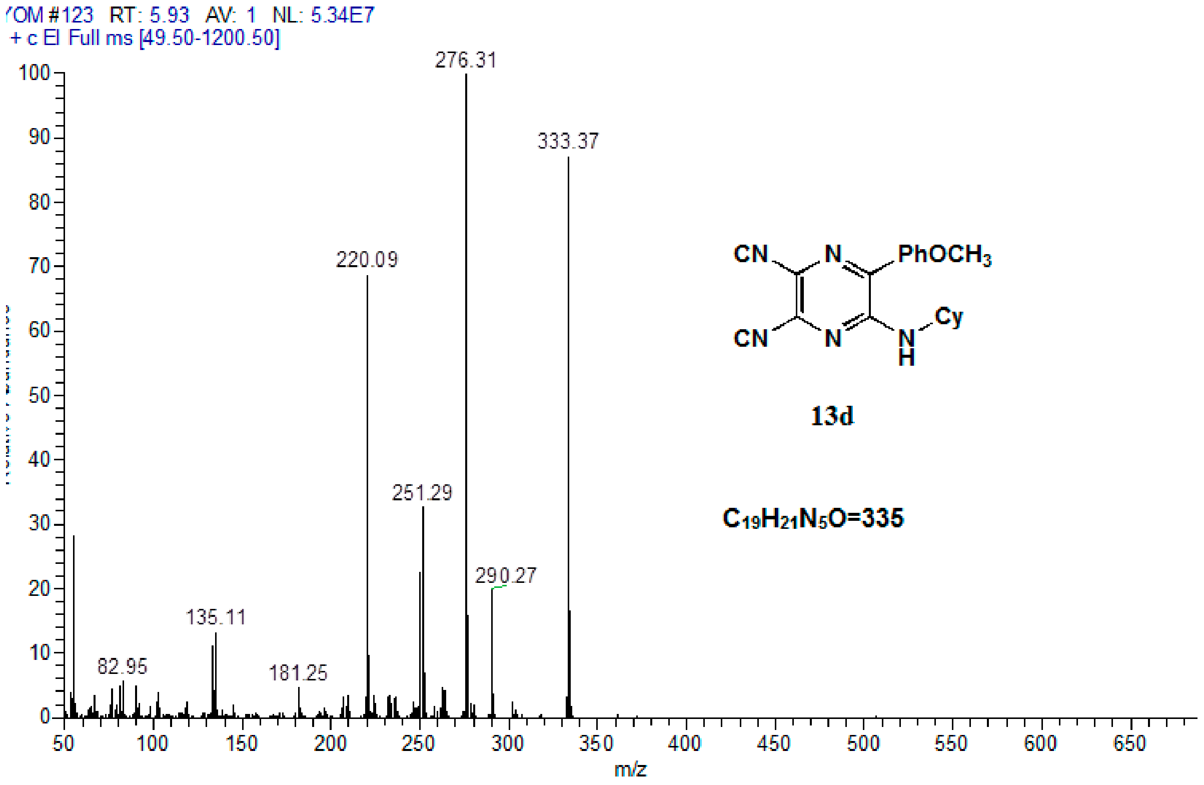
OM #123 RT: 5.93 AV: 1 NL: 5.34E7
+ c EI Full ms [49.50-1200.50]
276.31
333.37
220.09
251.29
290.27
135.11
181.25
82.95
m/z
CN
N
PhOCH3
CN
N
N
H
Cy
13d
C19H21N5O=335

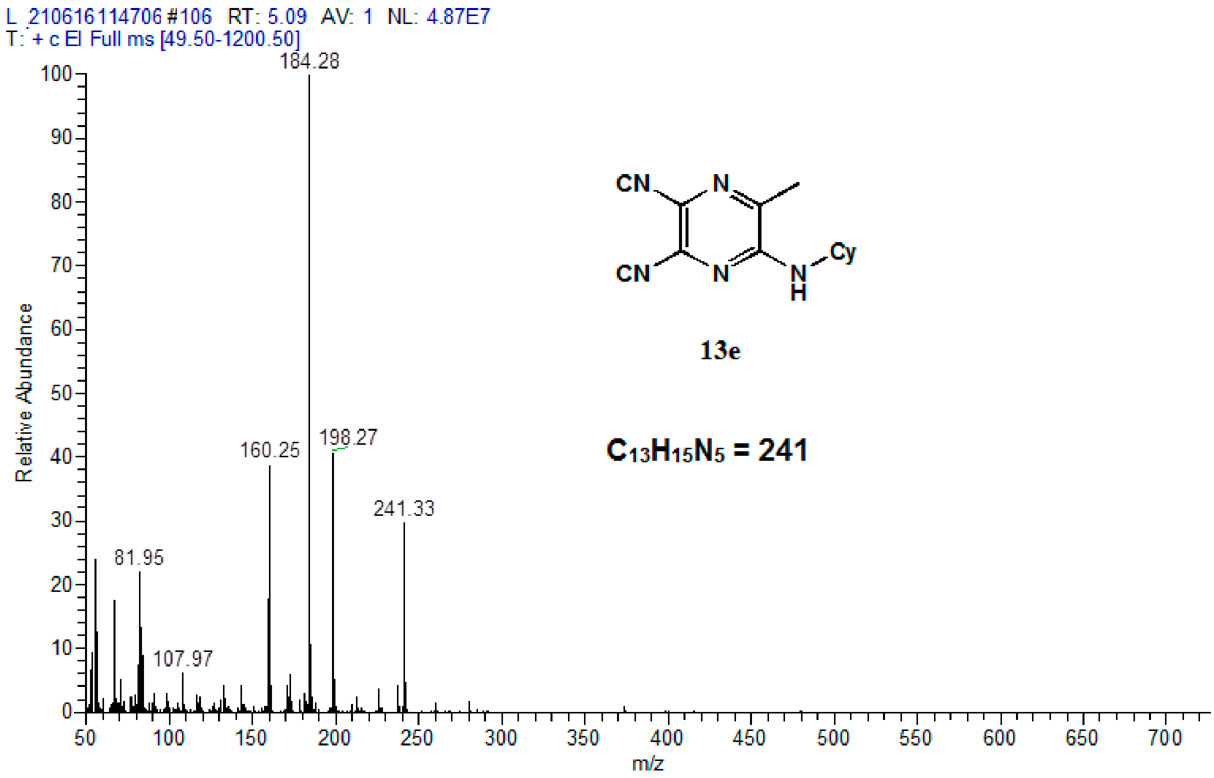
L_210616114706 #106 RT: 5.09 AV: 1 NL: 4.87E7
T: + c EI Full ms [49.50-1200.50]
Relative Abundance
m/z
184.28
160.25
198.27
241.33
81.95
107.97
CN
N
CN
N
N
H
Cy
13e
C13H15N5 = 241

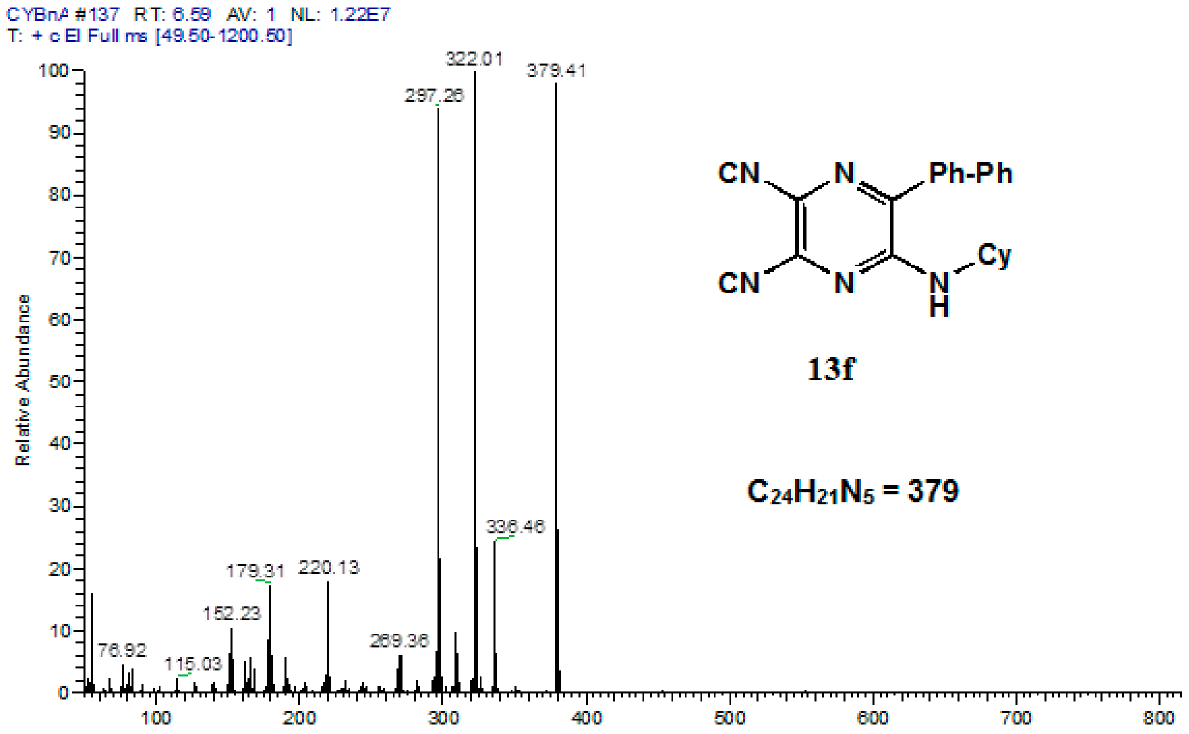
CYBnA #137 RT: 6.59 AV: 1 NL: 1.22E7
T: + c EI Full ms [49.50-1200.50]
Relative Abundance
322.01
379.41
297.26
336.46
179.31
220.13
152.23
269.36
76.92
115.03
CN
N
Ph-Ph
CN
N
N
H
Cy
13f
C24H21N5 = 379

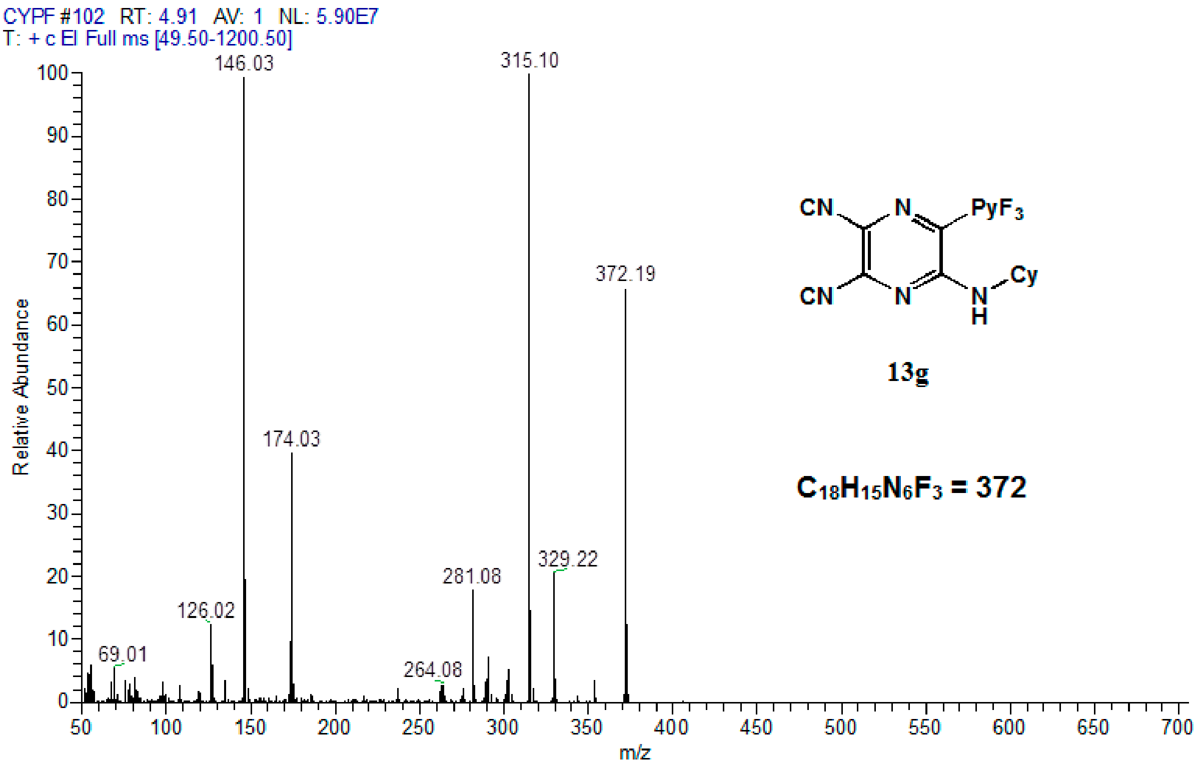
CYPF #102 RT: 4.91 AV: 1 NL: 5.90E7
T: + c EI Full ms [49.50-1200.50]
Relative Abundance
m/z
146.03
315.10
372.19
174.03
329.22
281.08
126.02
69.01
264.08
CN
N
PyF3
Cy
H
13g
C18H15N6F3 = 372

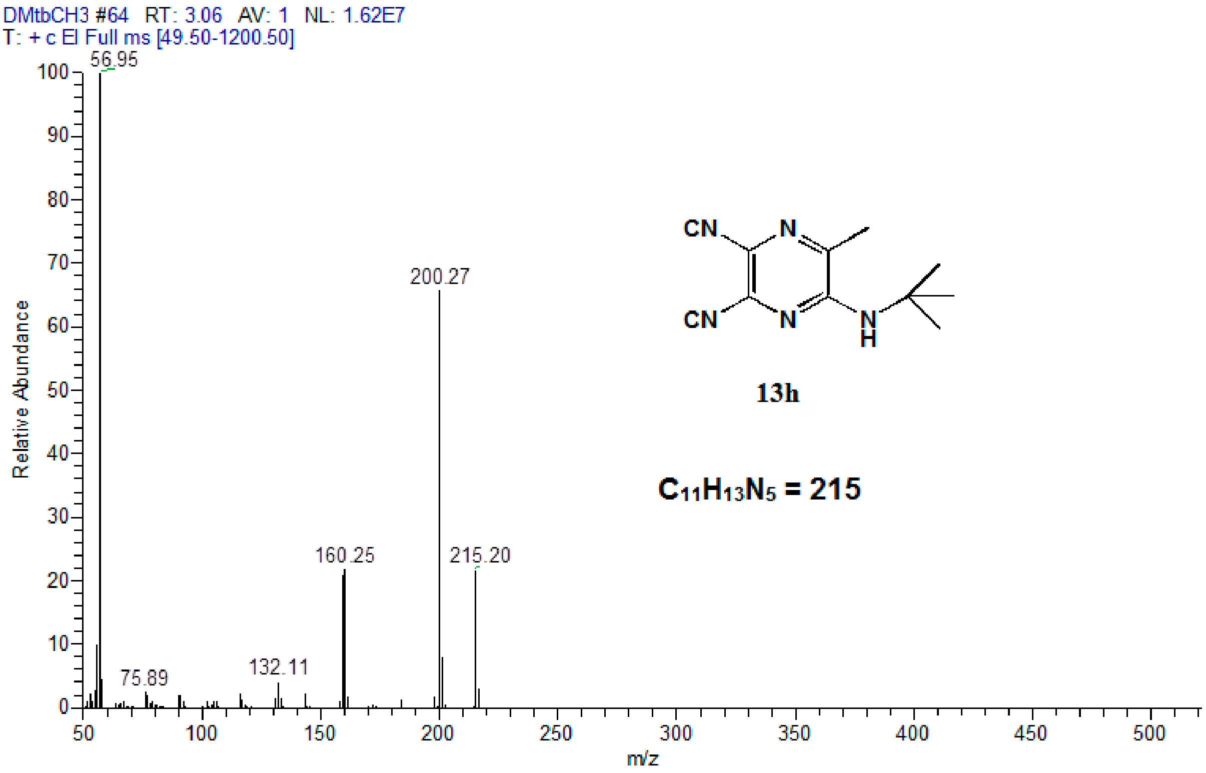
DMtbCH3 #64 RT: 3.06 AV: 1 NL: 1.62E7
T: + c EI Full ms [49.50-1200.50]
Relative Abundance
m/z
56.95
200.27
160.25
215.20
132.11
75.89
CN
N
H
13h
C11H13N5 = 215

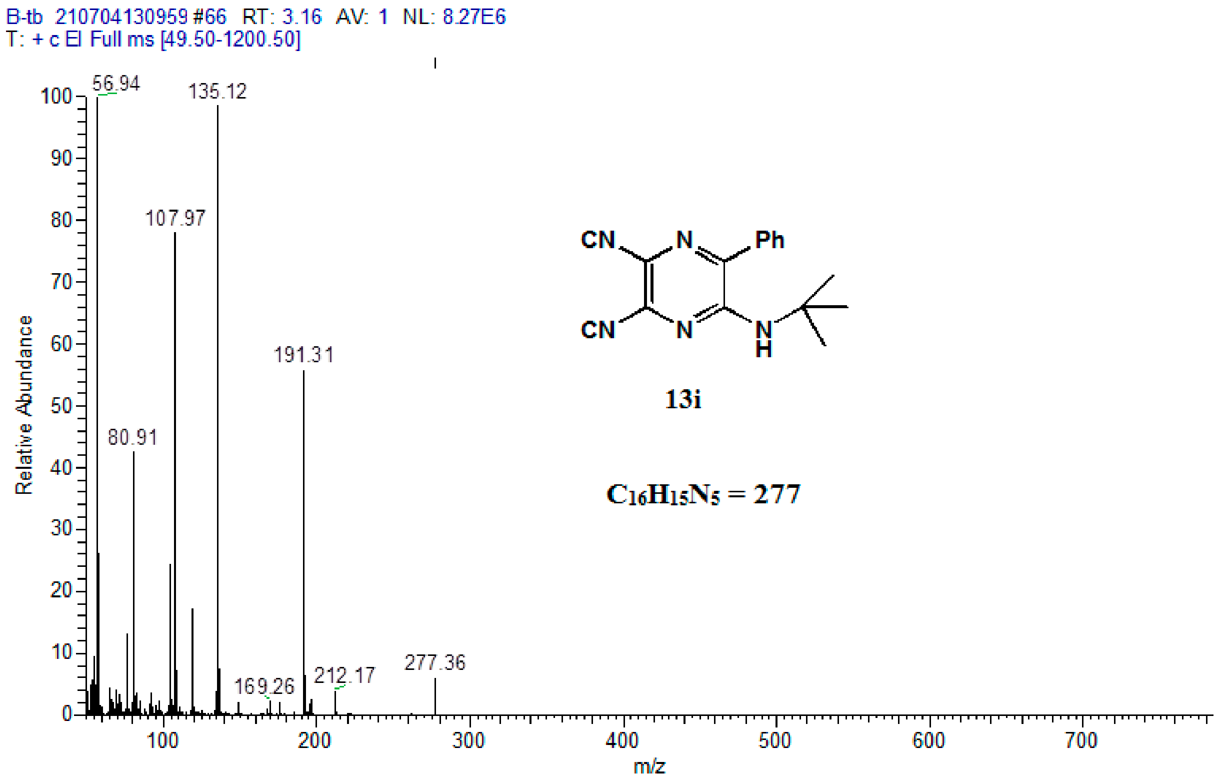
B-tb 210704130959 #66 RT: 3.16 AV: 1 NL: 8.27E6
T: + c EI Full ms [49.50-1200.50]
Relative Abundance
m/z
56.94
135.12
107.97
191.31
80.91
169.26
212.17
277.36
CN
N
Ph
CN
N
N
H
13i
C16H15N5 = 277

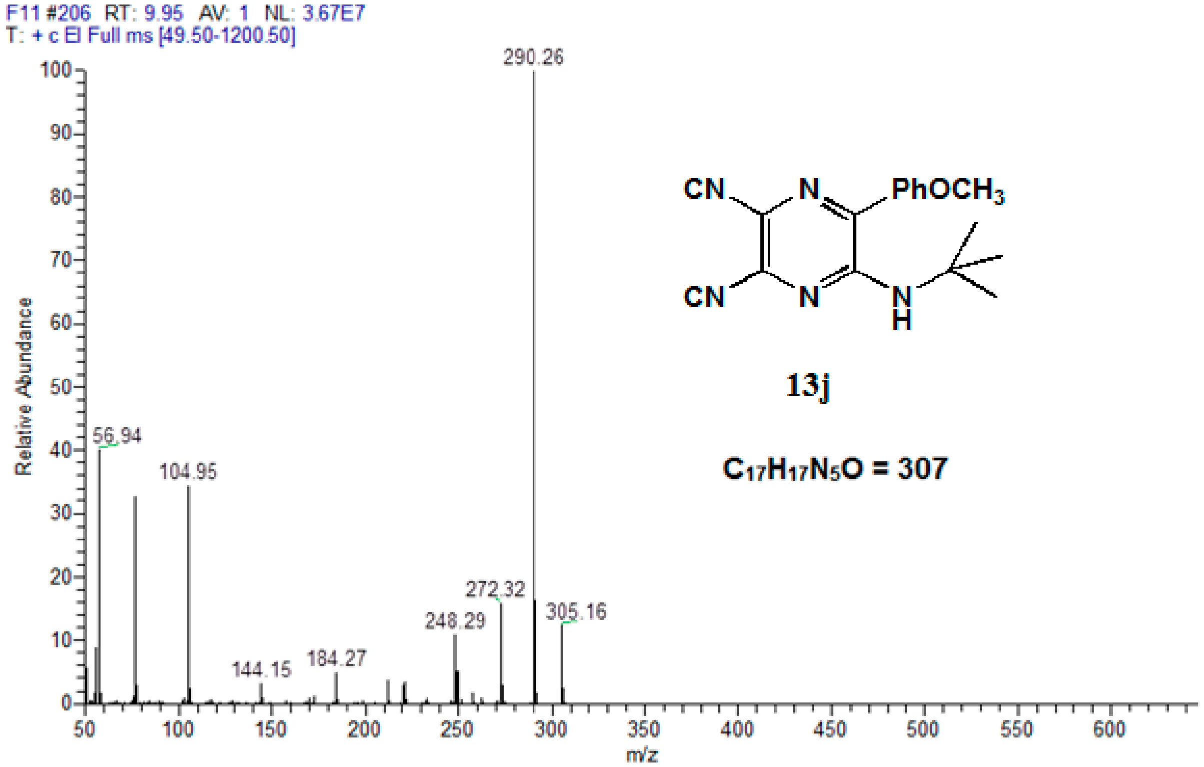
F11 #206 RT: 9.95 AV: 1 NL: 3.67E7
T: + c EI Full ms [49.50-1200.50]
Relative Abundance
m/z
290.26
56.94
104.95
272.32
248.29
305.16
144.15
184.27
CN
N
PhOCH3
CN
N
N
H
13j
C17H17N5O = 307

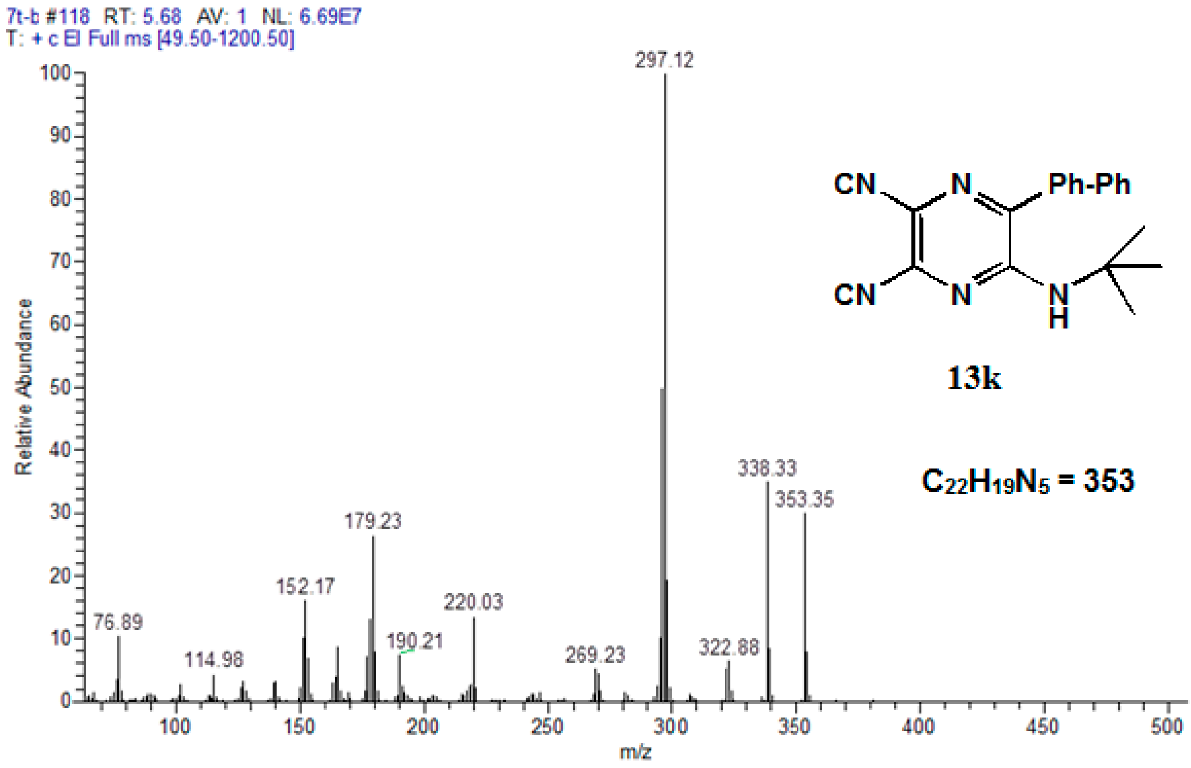
7t-b #118 RT: 5.68 AV: 1 NL: 6.69E7
T: + c EI Full ms [49.50-1200.50]
297.12
179.23
152.17
220.03
76.89
114.98
190.21
269.23
322.88
338.33
353.35
Relative Abundance
m/z
CN
N
Ph-Ph
CN
N
N
H
13k
C22H19N5 = 353

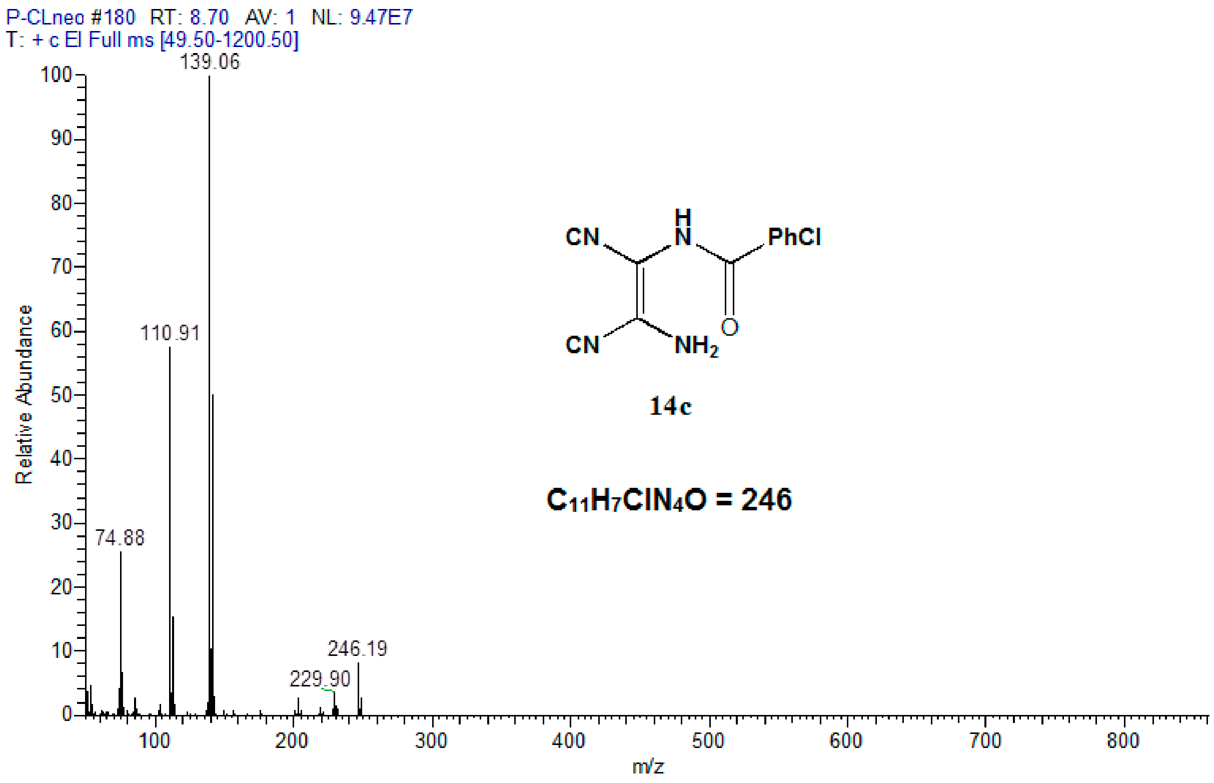
P-CLneo #180 RT: 8.70 AV: 1 NL: 9.47E7
T: + c EI Full ms [49.50-1200.50]
139.06
110.91
74.88
246.19
229.90
Relative Abundance
m/z
CN
H
N
PhCl
CN
NH2
O
14c
C11H7ClN4O = 246

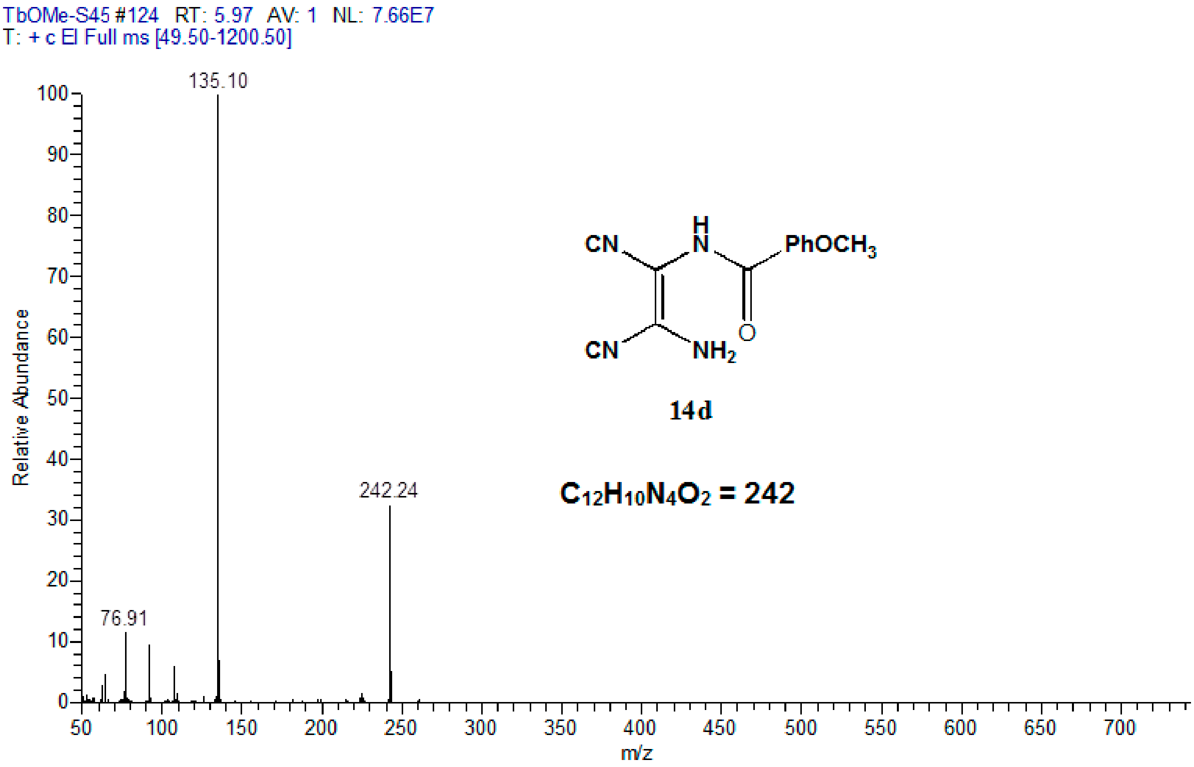
TbOMe-S45 #124 RT: 5.97 AV: 1 NL: 7.66E7
T: + c EI Full ms [49.50-1200.50]
135.10
242.24
76.91
Relative Abundance
m/z
CN
H
N
PhOCH3
CN
NH2
O
14d
C12H10N4O2 = 242

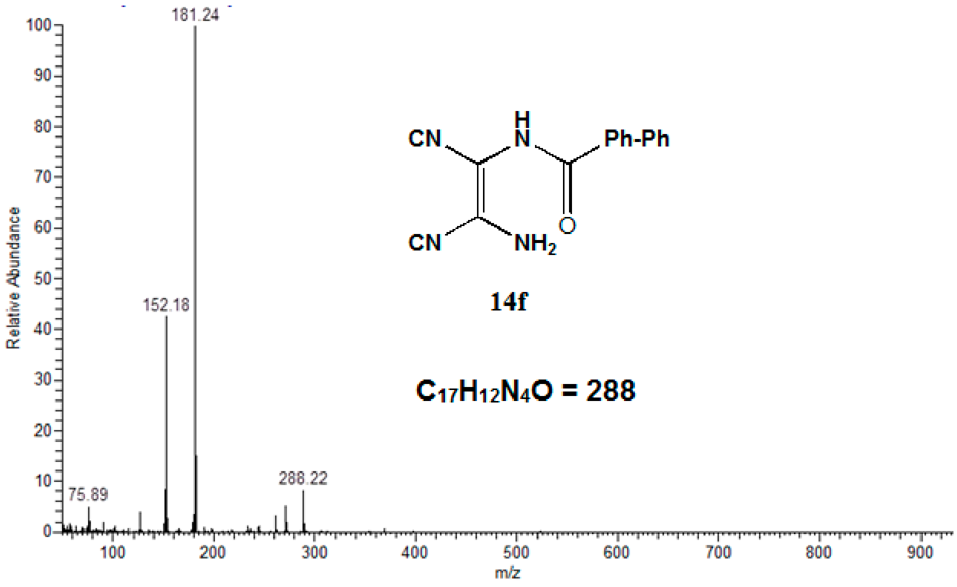
181.24
152.18
288.22
75.89
Relative Abundance
m/z
CN
H
N
Ph-Ph
CN
NH2
O
14f
C17H12N4O = 288

HRMS

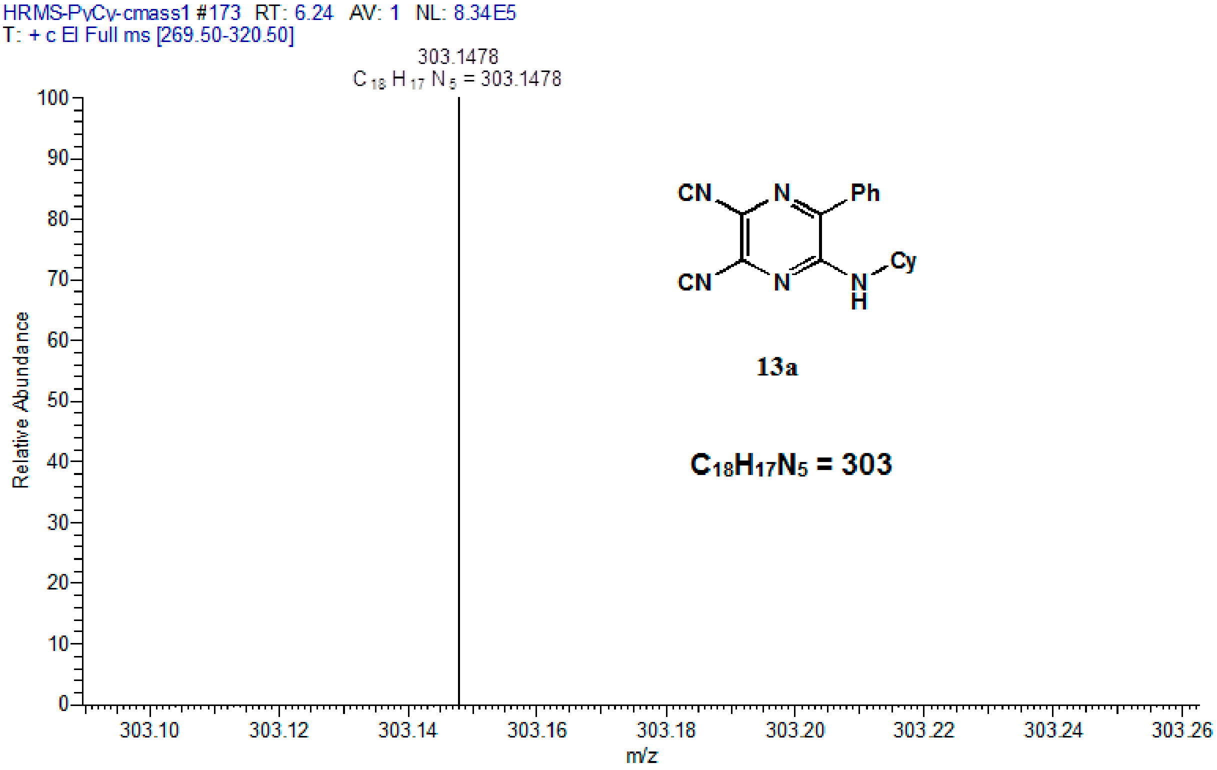

HRMS-PyCy-cmass1 #173 RT: 6.24 AV: 1 NL: 8.34E5
T: + c EI Full ms [269.50-320.50]
303.1478
C 18 H 17 N 5 = 303.1478
Relative Abundance
m/z
CN
CN
N
N
Ph
N
H
Cy
13a
C18H17N5 = 303

HRMS-MCY-cmass1 #89 RT: 7.62 AV: 1 NL: 2.60E2
T: + c EI Full ms [269.50-350.50]

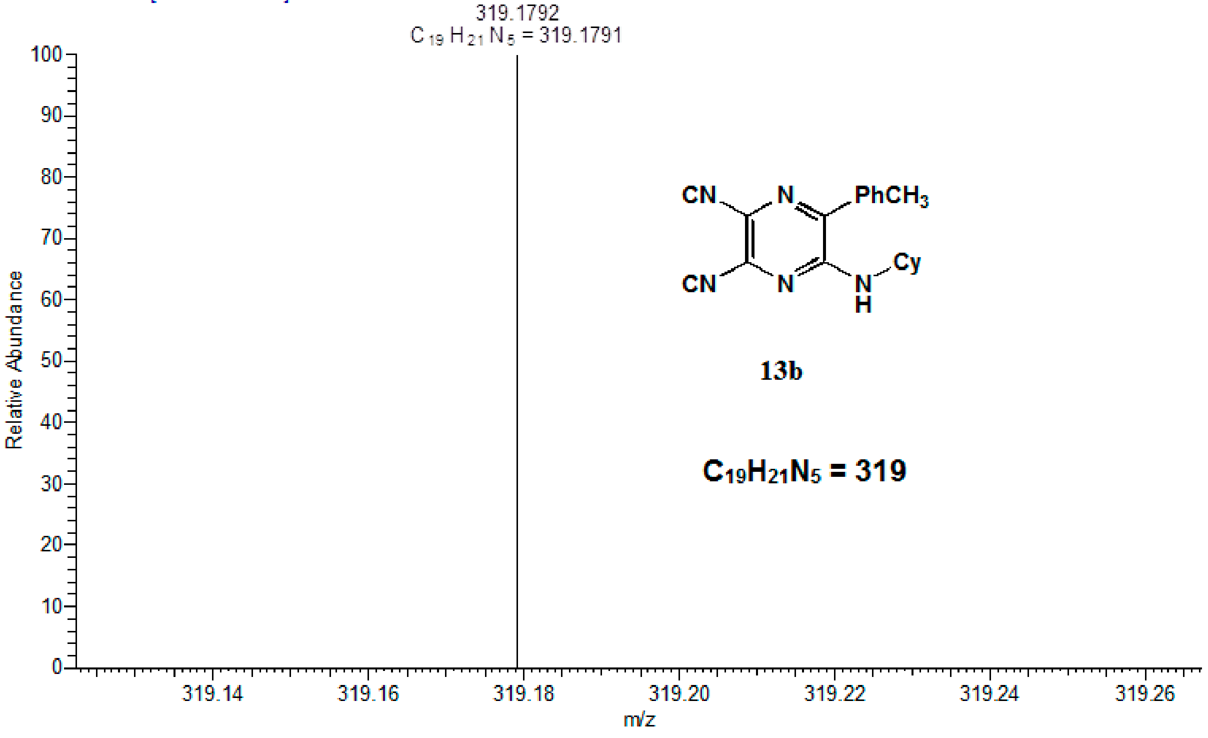

HRMS-PhCl-cmass1 #84 RT: 6.41 AV: 1 NL: 7.22E3
T: + c EI Full ms [299.50-370.50]

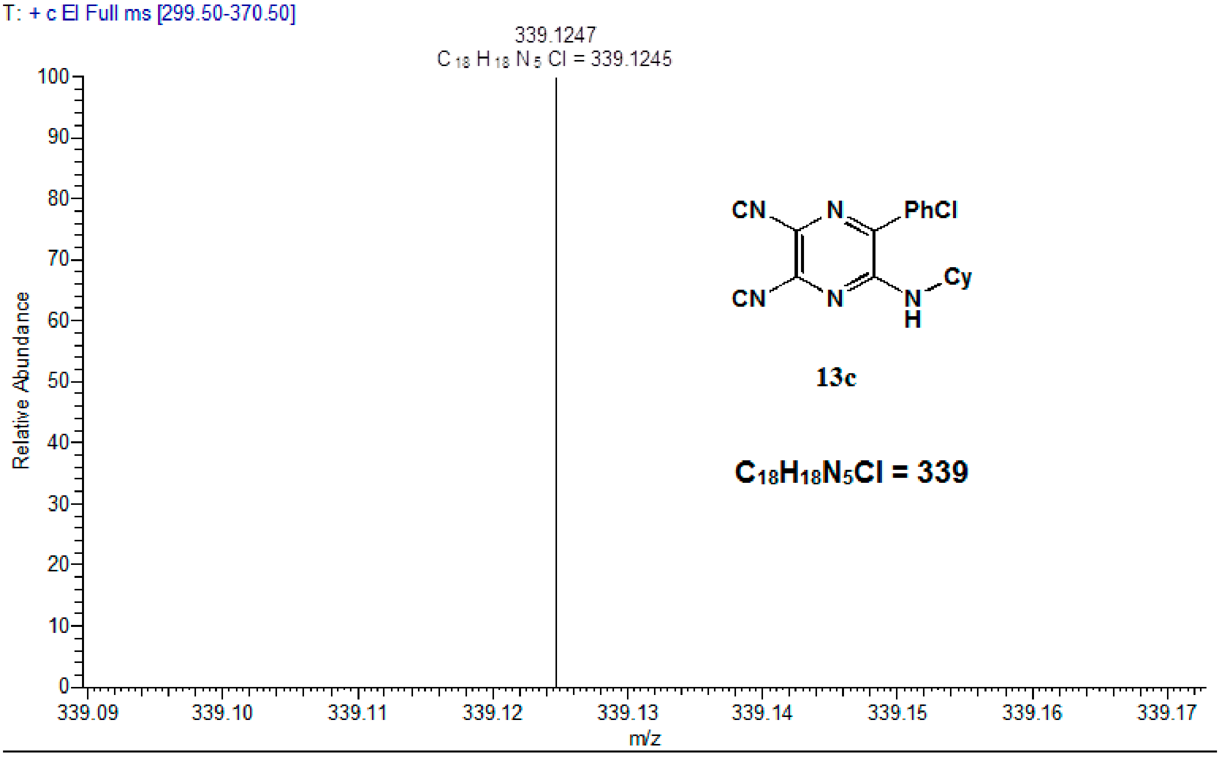

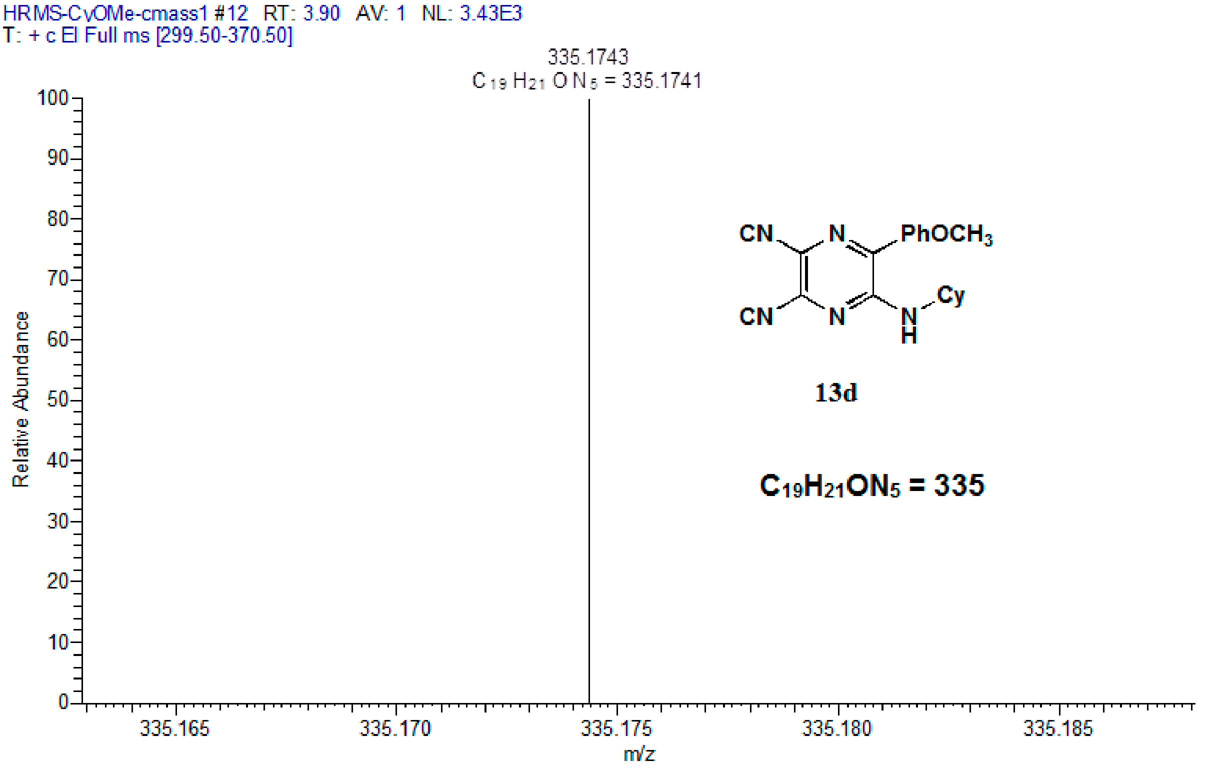
HRMS-CyOMe-cmass1 #12 RT: 3.90 AV: 1 NL: 3.43E3
T: + c EI Full ms [299.50-370.50]
335.1743
C 19 H 21 O N 5 = 335.1741
Relative Abundance
m/z
CN
N
PhOCH3
CN
N
N
H
Cy
13d
C19H21ON5 = 335

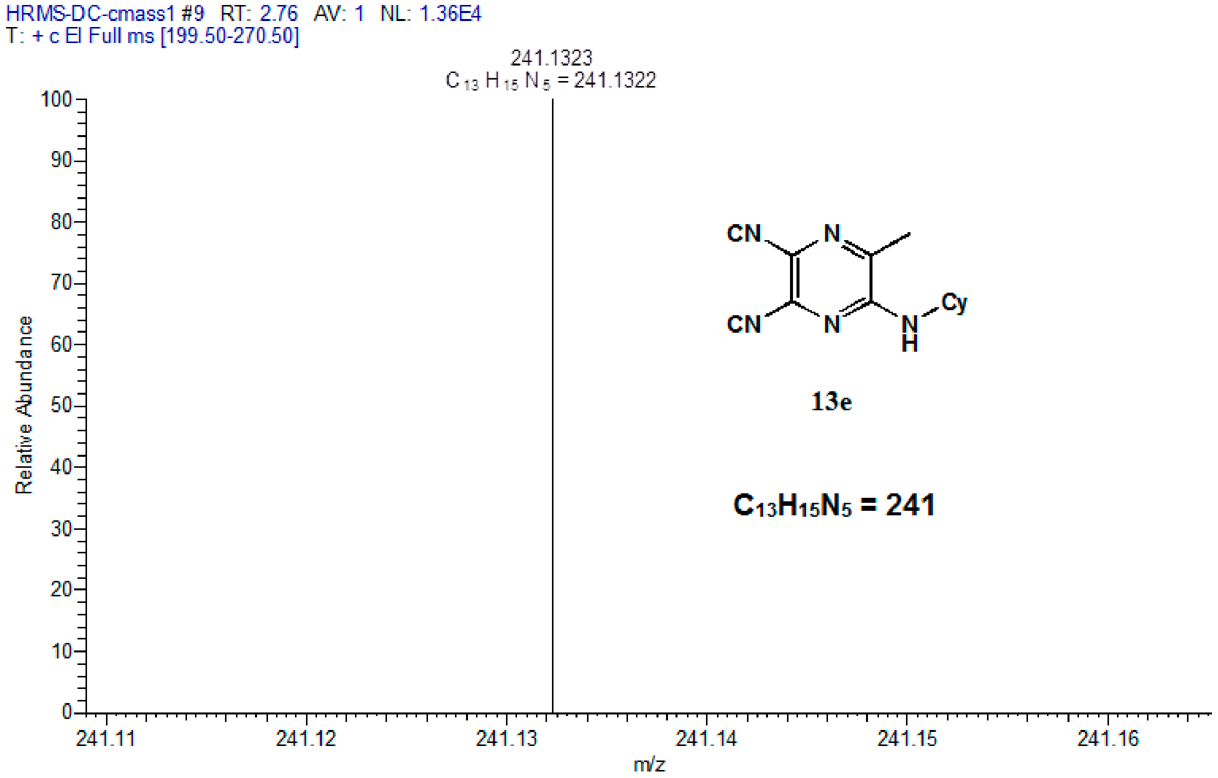
HRMS-DC-cmass1 #9 RT: 2.76 AV: 1 NL: 1.36E4
T: + c EI Full ms [199.50-270.50]
241.1323
C 13 H 15 N 5 = 241.1322
Relative Abundance
m/z
CN
CN
N
N
N
H
Cy
13e
C13H15N5 = 241

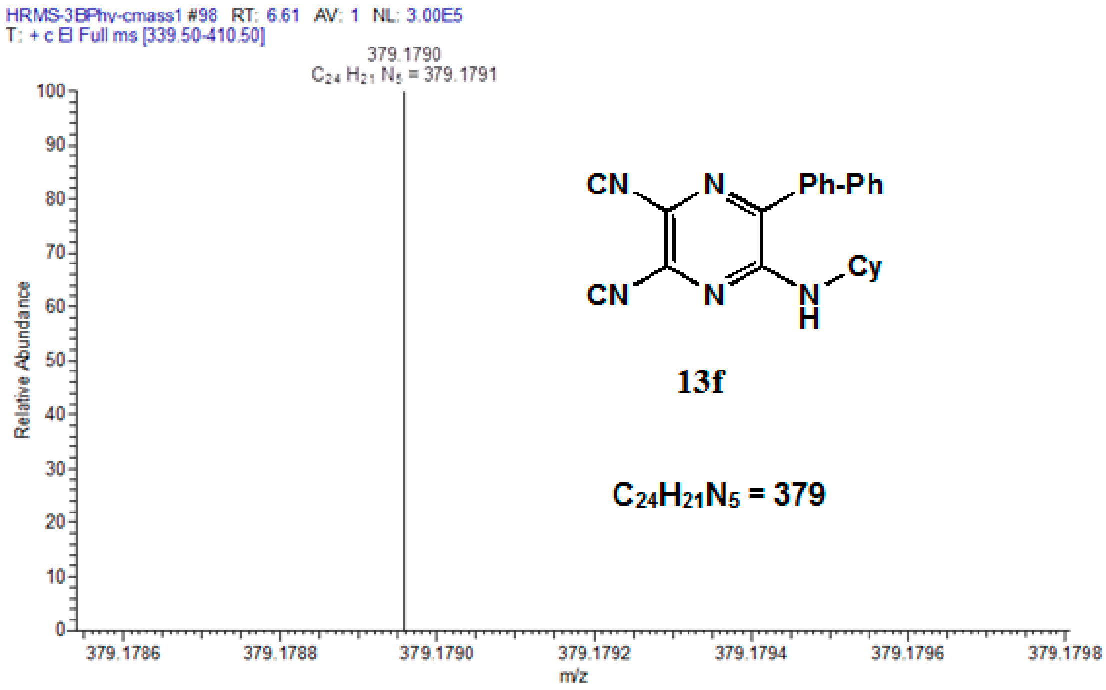
HRMS-3BPhy-cmass1 #98 RT: 6.61 AV: 1 NL: 3.00E5
T: + c EI Full ms [339.50-410.50]
379.1790
C24 H21 N5 = 379.1791
Relative Abundance
m/z
CN
CN
N
N
Ph-Ph
N
H
Cy
13f
C24H21N5 = 379

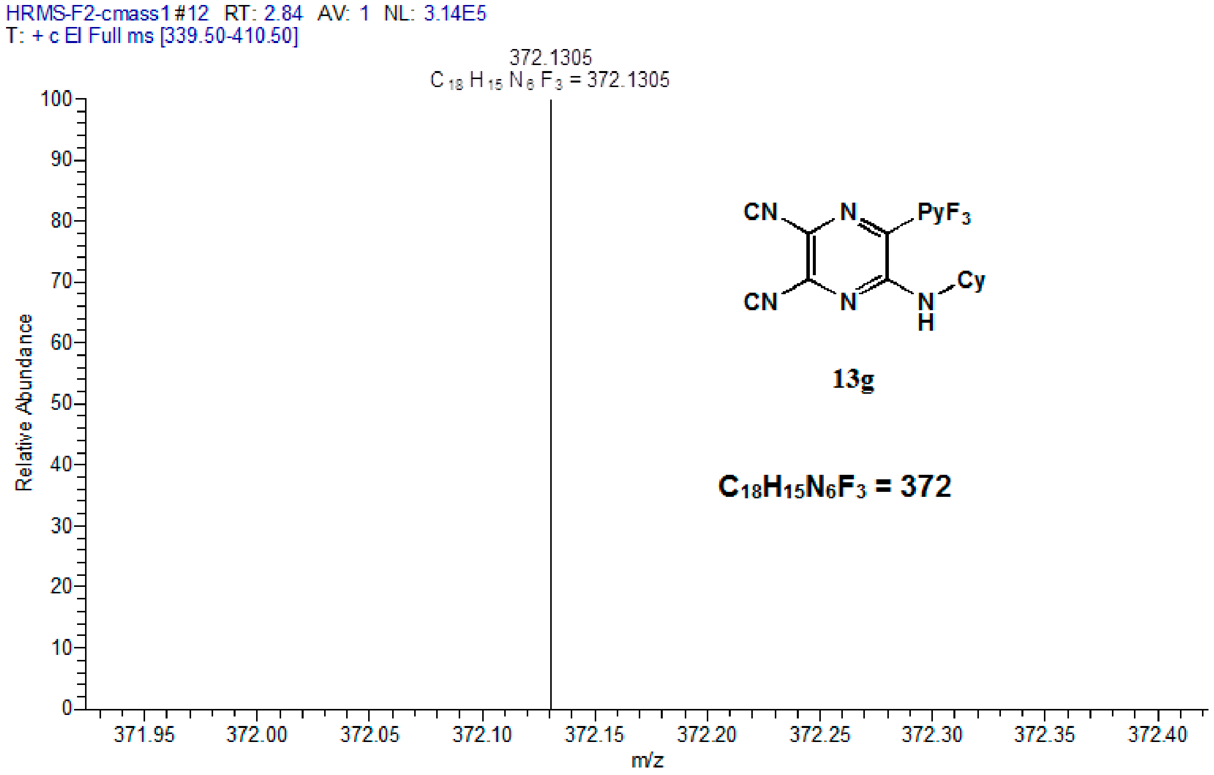
HRMS-F2-cmass1 #12 RT: 2.84 AV: 1 NL: 3.14E5
T: + c EI Full ms [339.50-410.50]
372.1305
C 18 H 15 N 6 F 3 = 372.1305
Relative Abundance
m/z
CN
CN
N
N
PyF3
N
H
Cy
13g
C18H15N6F3 = 372

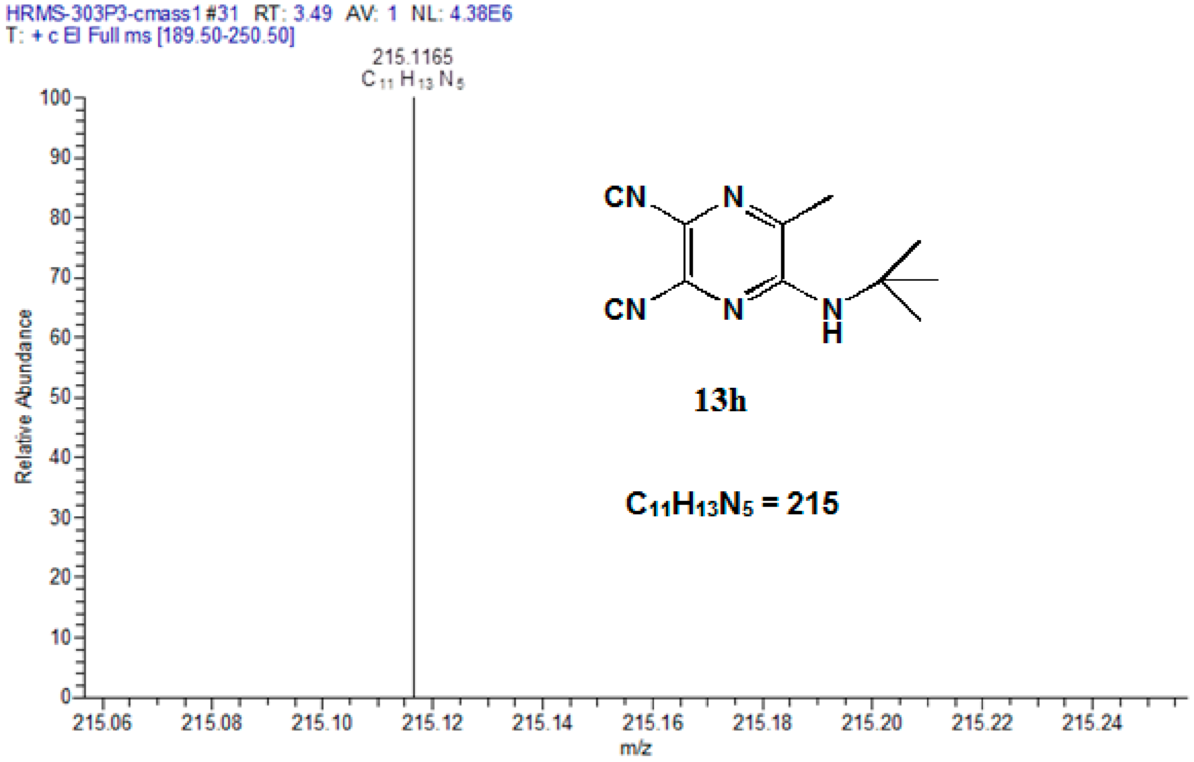
HRMS-303P3-cmass1 #31 RT: 3.49 AV: 1 NL: 4.38E6
T: + c EI Full ms [189.50-250.50]
215.1165
C 11 H 13 N 5
Relative Abundance
m/z
CN
CN
N
N
N
H
13h
C11H13N5 = 215

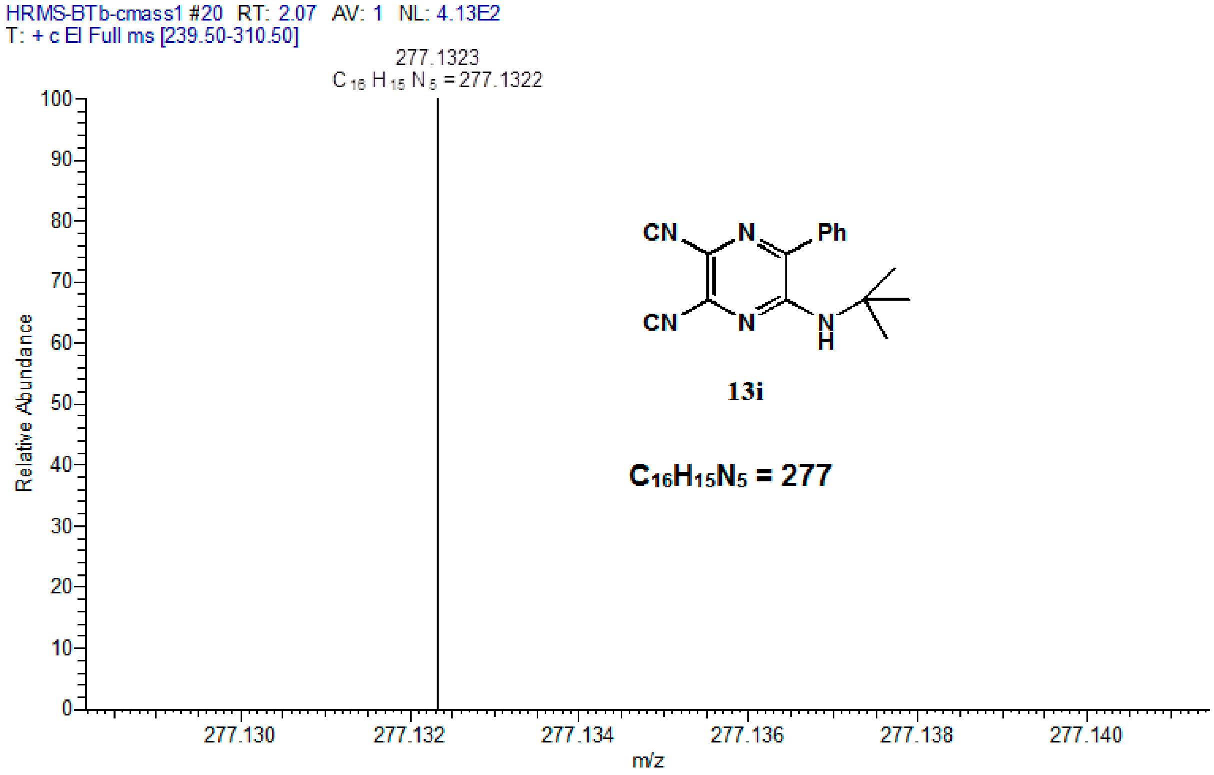

HRMS-BTb-cmass1 #20 RT: 2.07 AV: 1 NL: 4.13E2
T: + c EI Full ms [239.50-310.50]
277.1323
C 16 H 15 N 5 = 277.1322
Relative Abundance
m/z
CN
N
Ph
CN
N
N
H
13i
C16H15N5 = 277

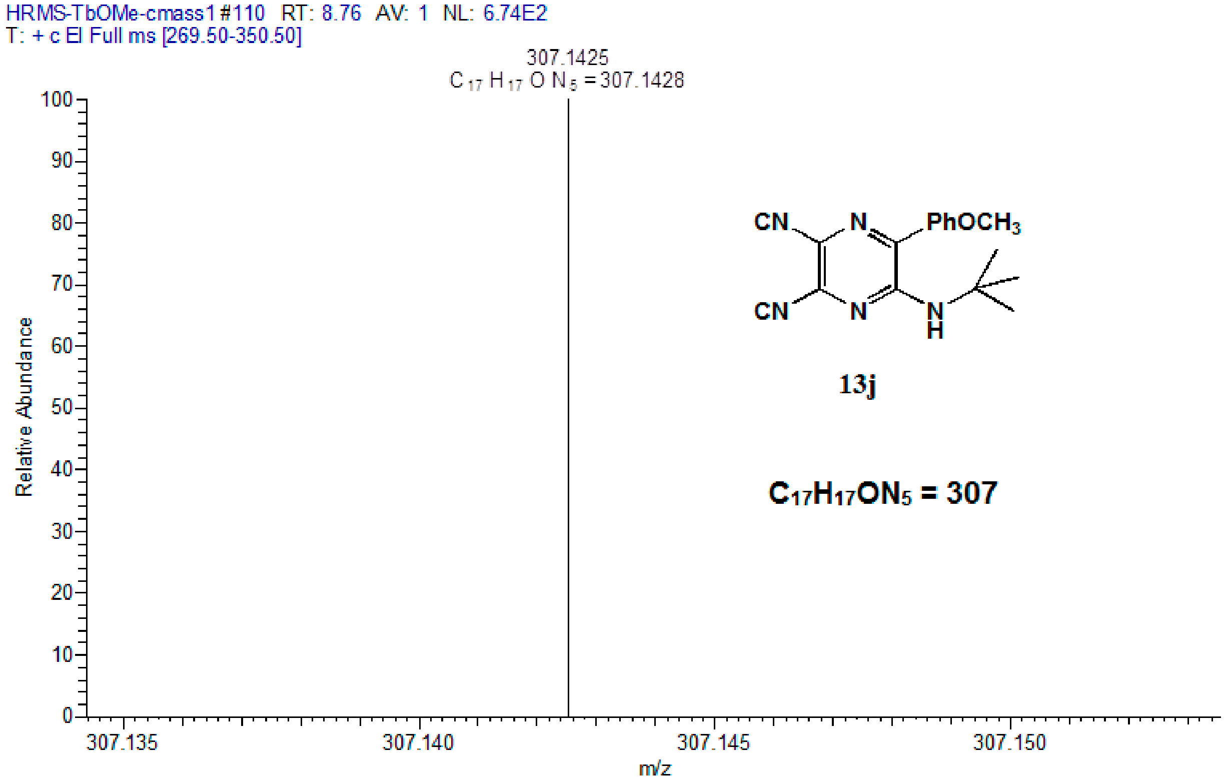

HRMS-TbOMe-cmass1 #110 RT: 8.76 AV: 1 NL: 6.74E2
T: + c EI Full ms [269.50-350.50]
307.1425
C 17 H 17 O N 5 = 307.1428
Relative Abundance
m/z
CN
N
PhOCH3
CN
N
N
H
13j
C17H17ON5 = 307

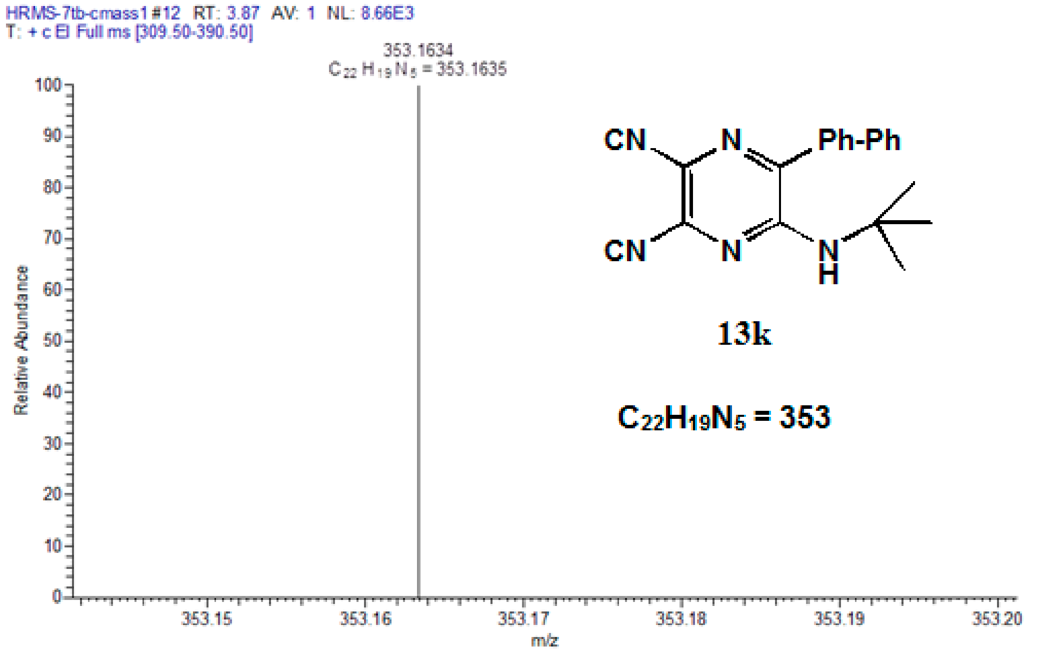
HRMS-7tb-cmass1 #12 RT: 3.87 AV: 1 NL: 8.66E3
T: + c EI Full ms [309.50-390.50]
353.1634
C 22 H 19 N 5 = 353.1635
Relative Abundance
m/z
CN
N
Ph-Ph
CN
N
N
H
13k
C22H19N5 = 353

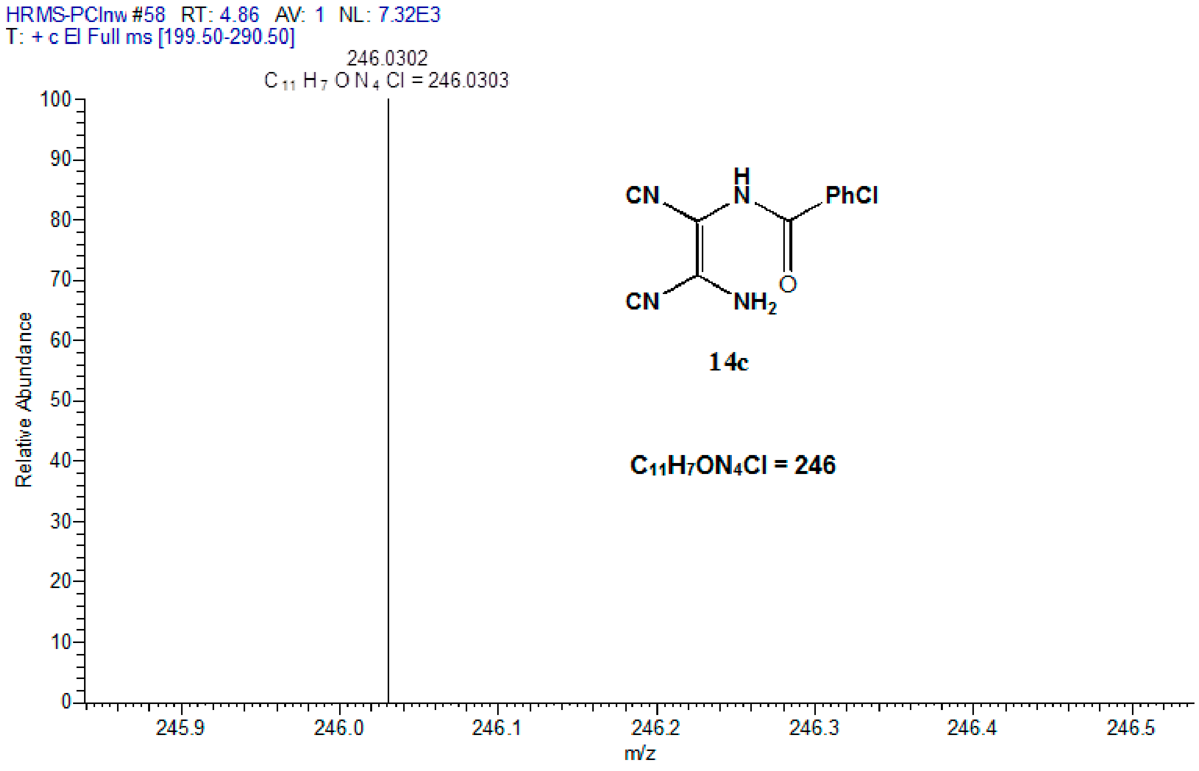
HRMS-PClnw #58 RT: 4.86 AV: 1 NL: 7.32E3
T: + c EI Full ms [199.50-290.50]
246.0302
C 11 H 7 O N 4 Cl = 246.0303
Relative Abundance
m/z
CN
H
N
PhCl
CN
NH2
O
14c
C11H7ON4Cl = 246

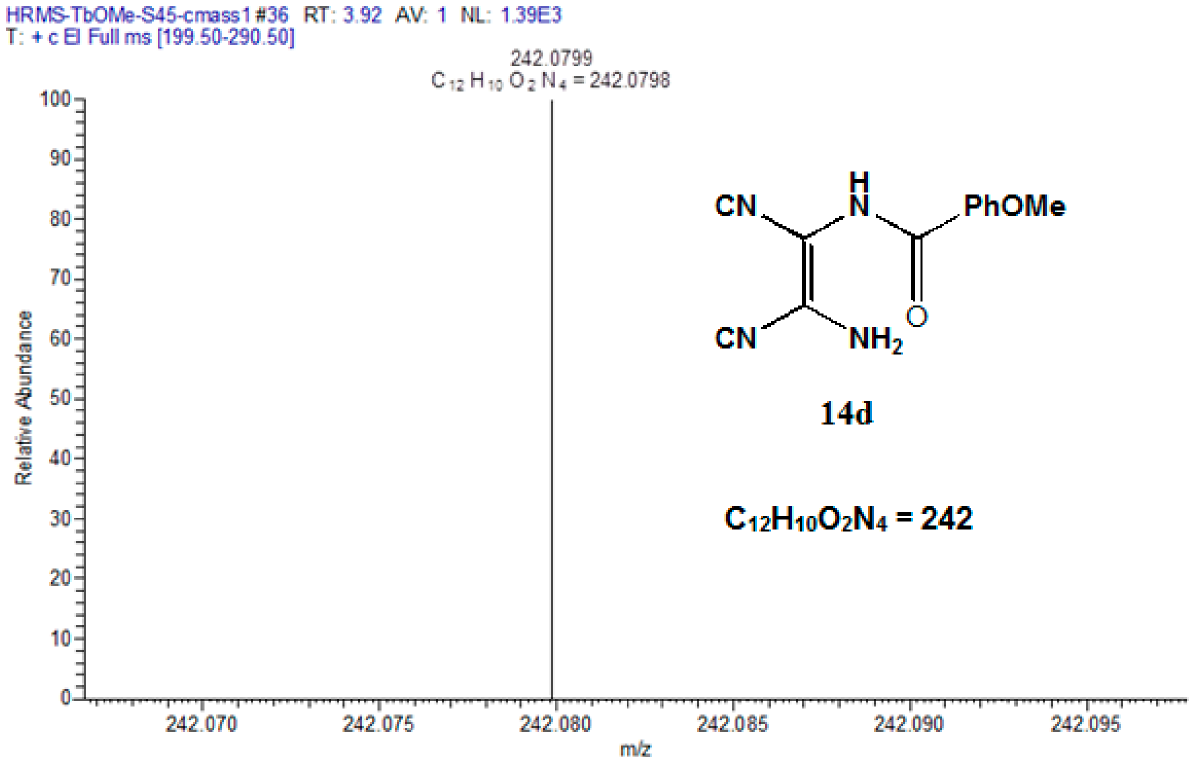
HRMS-TbOMe-S45-cmass1 #36 RT: 3.92 AV: 1 NL: 1.39E3
T: + c EI Full ms [199.50-290.50]
242.0799
C 12 H 10 O 2 N 4 = 242.0798
Relative Abundance
m/z
CN
H
N
PhOMe
CN
NH2
O
14d
C12H10O2N4 = 242

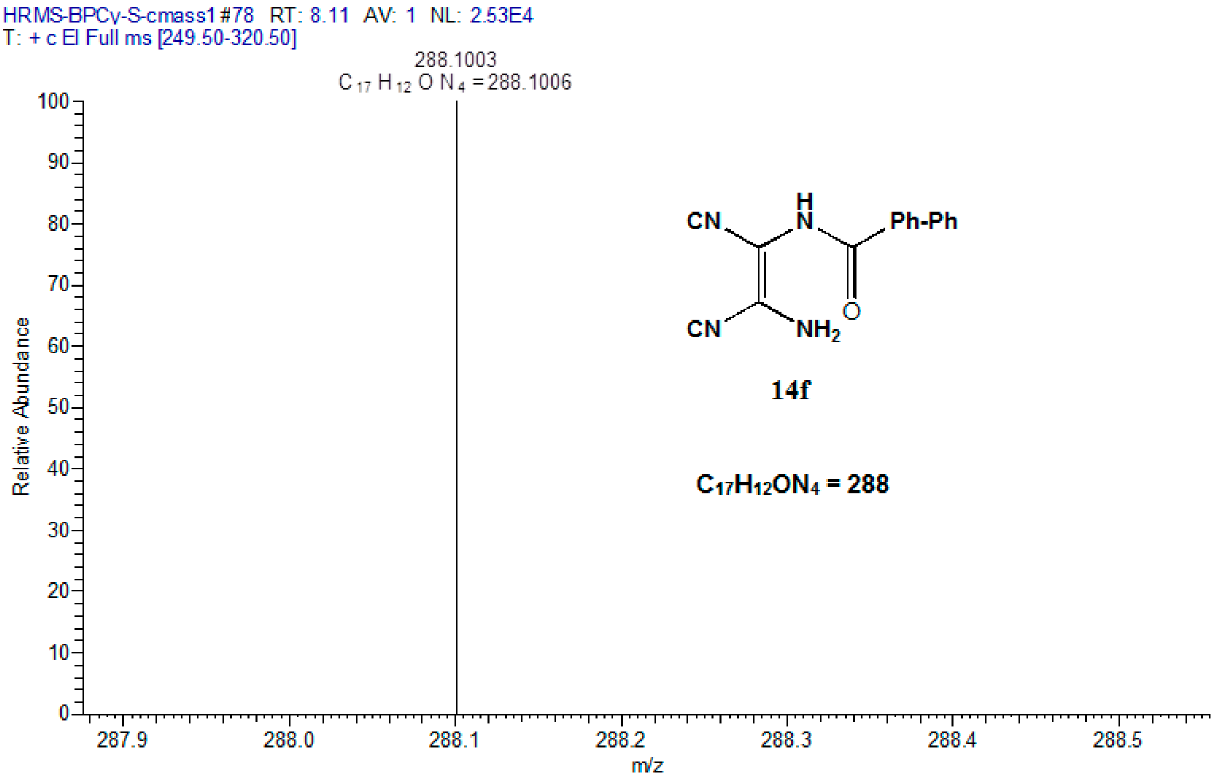
HRMS-BPCy-S-cmass1 #78 RT: 8.11 AV: 1 NL: 2.53E4
T: + c EI Full ms [249.50-320.50]
288.1003
C 17 H 12 O N 4 = 288.1006
Relative Abundance
m/z
CN
CN
H
N
NH2
O
Ph-Ph
14f
C17H12ON4 = 288

Single crystal X- diffraction analysis of 13a, 13e, 13f & 13k

Experimental

The single crystal data collection was made either on Bruker X8 prospector diffractometer by Cu-Kα radiation or on Rigaku Rapid II diffractometer by Mo-Kα radiation at room temperature. In the former case the reflection frames were integrated with the Bruker SAINT Software package using a narrow-frame algorithm. Finally, the structure was solved using the Bruker SHELXTL Software Package and refined using SHELXL-2017/1. The data collected using the Rigaku diffractometer was processed by 'Crystalclear' software package. The structure was then solved by direct methods by 'CrystalStructure' crystallographic software package and the refinement was performed using SHELXL-2017/1. All non-hydrogen atoms were refined anisotropically.

The summary of the various crystallographic parameters of the 5 crystal samples discussed in the present study are provided in the **Table S1** & **Table S2**.

Table S1. Summary on the nature and various crystallographic parameters of **13a, 13e, 13f** & **13k**

**Crystal sample**	**13a**	**13e**
Chemical formula	$C_{18}H_{17}N_5$	$C_{13}H_{15}N_5$
M_r	303.36	241.30
Crystal system, space group	Monoclinic, *P*2$_1$/*c*	Orthorhombic, *Pnma*
Temperature (K)	296	296
a, *b*, *c* (Å)	12.5902 (4), 7.3148 (3), 18.7251 (7)	22.2572 (18), 6.7423 (6), 8.8558 (7)
α, β, γ (°)	90, 106.991 (2), 90	90, 90, 90
V (Å^3)	1649.21 (11)	1328.94 (19)
Z	4	4
Radiation type	Cu *K*α	Cu *K*α
μ (mm^{-1})	0.61	0.62
Crystal size (mm)	0.34 × 0.27 × 0.12	0.20 × 0.20 × 0.10
Diffractometer	Bruker *APEX*-II CCD	Bruker *APEX*-II CCD
Absorption correction	Multi-scan<br><br>*SADABS2016*/2 - Bruker AXS area detector scaling and absorption correction	Multi-scan<br><br>*SADABS2016*/2 - Bruker AXS area detector scaling and absorption correction

T_{min}, T_{max}	0.83, 0.92	0.88, 0.94
No. of measured, independent & observed [$I > 2\sigma(I)$] reflections	18753, 2907, 2085	5714, 1269, 1033
R_{int}	0.061	0.046
$(\sin \theta/\lambda)_{max}$ (Å^{-1})	0.596	0.595
$R[F^2 > 2\sigma(F^2)]$, $wR(F^2)$, S	0.048, 0.144, 1.01	0.058, 0.181, 1.10
No. of reflections	2907	1269
No. of parameters	208	110
H-atom treatment	Constrained	Mixed
$\Delta\rho_{max}$, $\Delta\rho_{min}$ (e Å^{-3})	0.11, −0.22	0.26, −0.20

Table S2. Summary on the nature and various crystallographic parameters of **13f** & **13k**

**Crystal sample**	**13f**	**13k**
Chemical formula	$C_{24}H_{21}N_5$	$C_{22}H_{19}N_5$
M_r	379.46	353.42
Crystal system, space group	Monoclinic, $P2_1/n$	Monoclinic, $P2_1/c$
Temperature (K)	296	296
a, b, c (Å)	11.8808 (4), 10.5852 (4), 17.3946 (7)	12.4754 (9), 10.2482 (7), 15.7377 (12)
α, β, γ (°)	90, 106.785 (3), 90	90, 104.935 (4), 90
V (Å^3)	2094.36 (14)	1944.1 (2)
Z	4	4
Radiation type	Cu $K\alpha$	Cu $K\alpha$
μ (mm^{-1})	0.58	0.59
Crystal size (mm)	0.22 × 0.14 × 0.11	0.16 × 0.15 × 0.11
Diffractometer	Bruker *APEX*-II CCD	Bruker *APEX*-II CCD
Absorption correction	Multi-scan<br><br>*SADABS2016*/2 - Bruker AXS area detector scaling and absorption correction	Multi-scan<br><br>*SADABS2016*/2 - Bruker AXS area detector scaling and absorption correction
T_{min}, T_{max}	0.88, 0.93	0.90, 0.94

No. of measured, independent & observed [$I > 2\sigma(I)$] reflections	10564, 3621, 2460	17845, 3335, 2140
R_{int}	0.045	0.062
$(\sin \theta/\lambda)_{max}$ (Å^{-1})	0.596	0.596
$R[F^2 > 2\sigma(F^2)]$, $wR(F^2)$, S	0.055, 0.165, 1.04	0.060, 0.194, 1.08
No. of reflections	3621	3335
No. of parameters	262	244
H-atom treatment	Constrained	Constrained
$\Delta\rho_{max}$, $\Delta\rho_{min}$ (e Å^{-3})	0.20, −0.18	0.16, −0.19

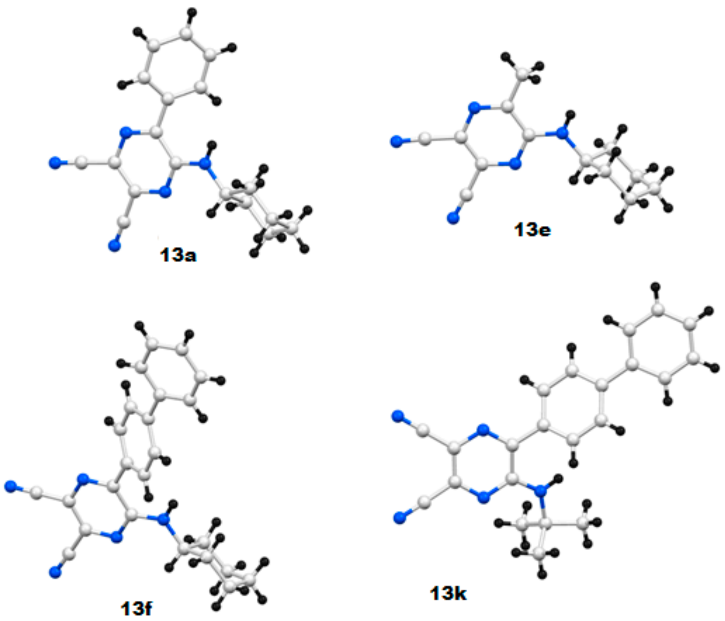

Figure S1. Crystal structure of **13a**, **13e, 13f and 13k**. Color code : gray-carbon; blue-nitrogen; yellow-flouring and black-hydrogen.

Supporting information

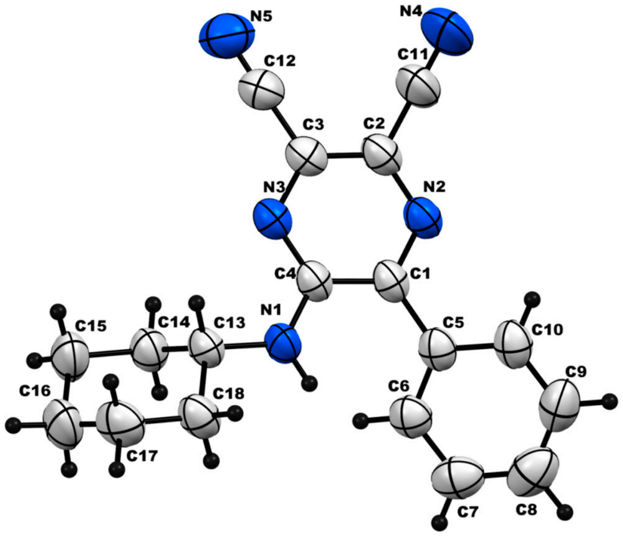

Figure S2. Crystal structure (Thermal ellipsoid representation) of **13a**.

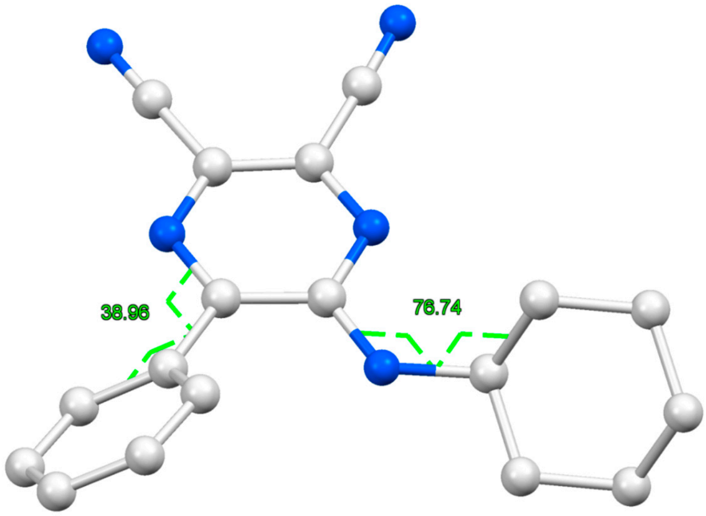

Figure S3. Orientation and the corresponding torsion angles of cyclohexyl and phenyl rings against the pyrazene ring in the crystal structure of the molecule **13a**.

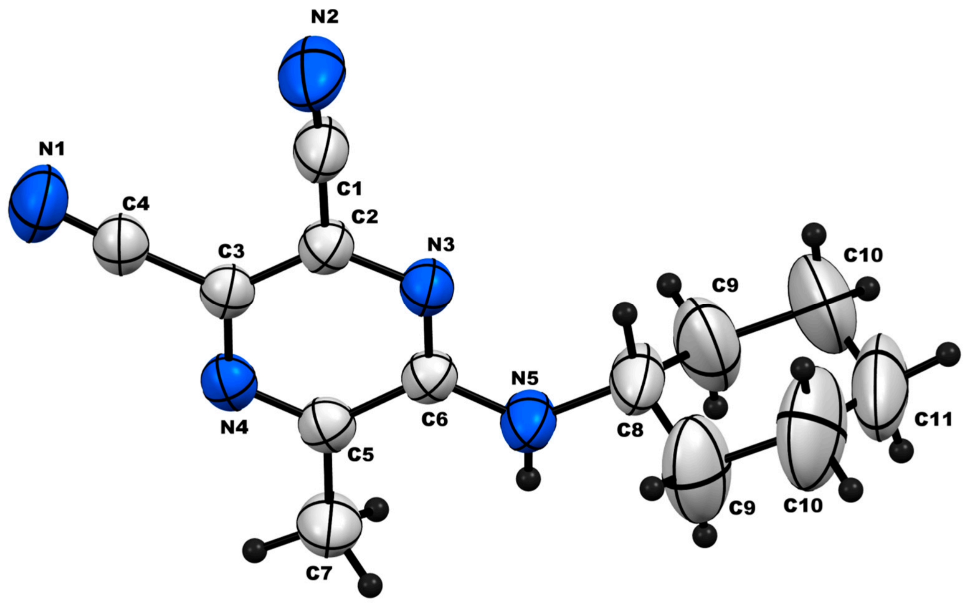

Figure S4. Crystal structure (Thermal ellipsoid representation) of **13e**.

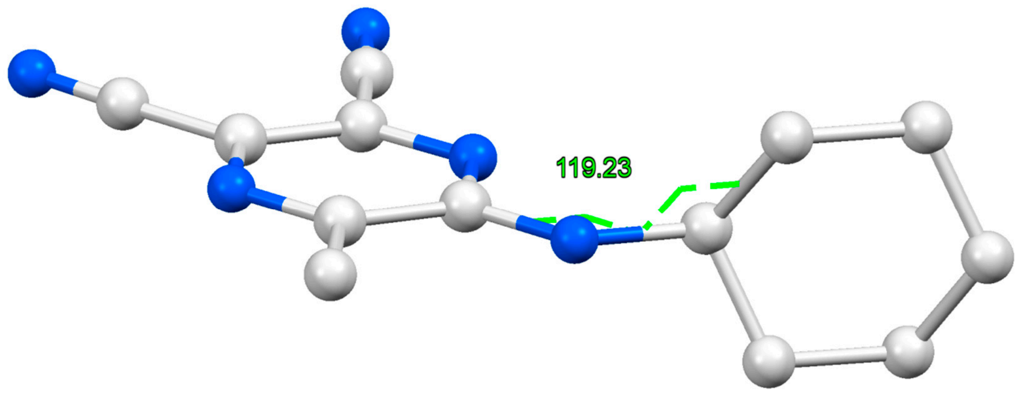

Figure S5. Orientation and the corresponding torsion angle of cyclohexyl ring against the pyrazene ring in the crystal structure of the molecule **13e**.

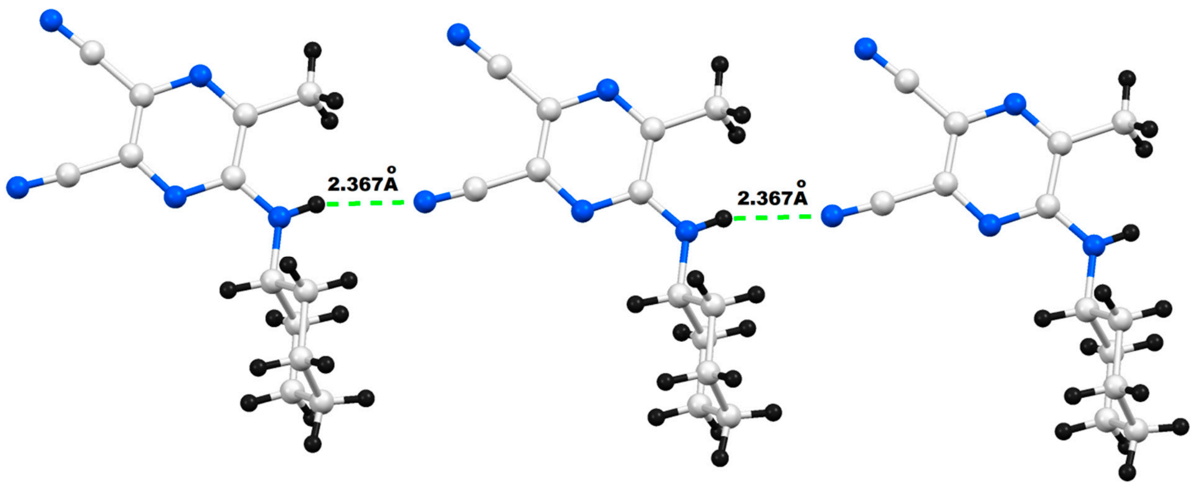

Figure S6. Intermolecular H-bonds experienced by **13e** molecules in its crystal network.

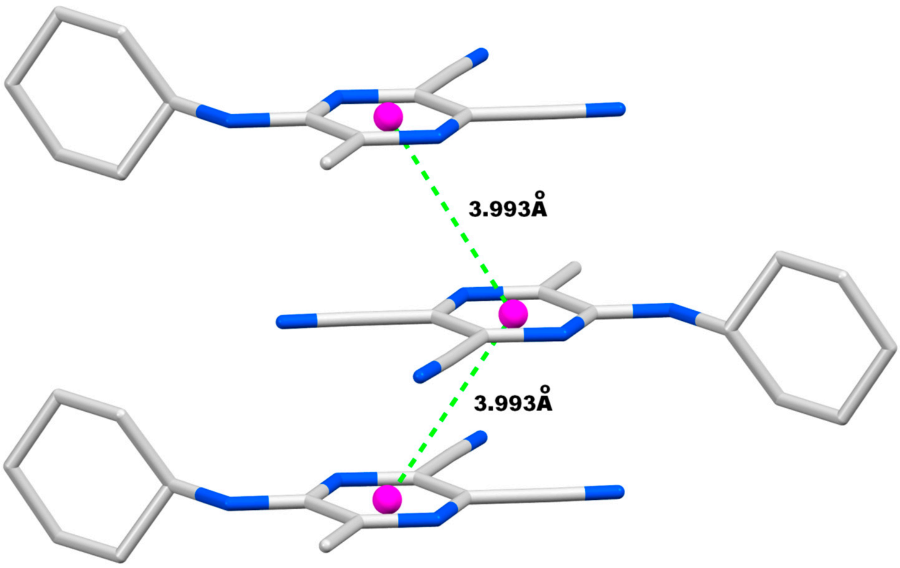

Figure S7. π - π interactions among **13e** molecules in their crystal network

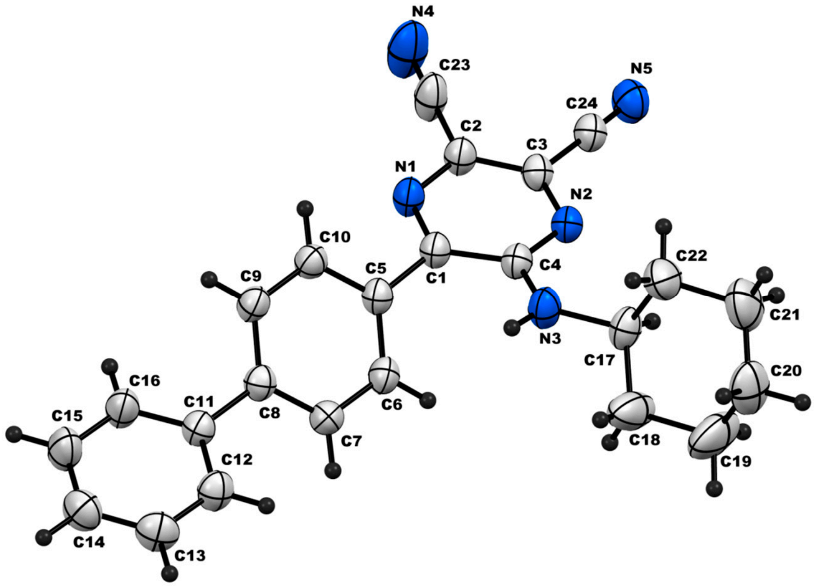

Figure S8. Crystal structure (Thermal ellipsoid representation) of **13f**.

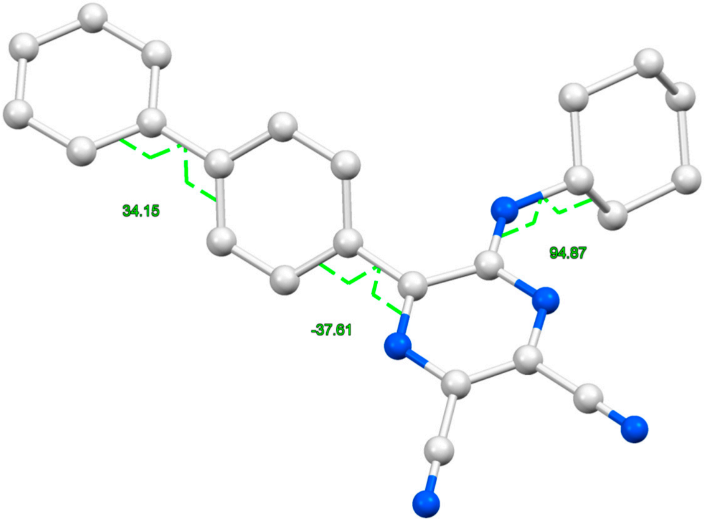

Figure S9. Orientation and the corresponding torsion angles of cyclohexyl and phenyl ring against the pyrazene ring along with the phenyl-phenyl orientation of the bi-phenyl moiety in the crystal structure of the molecule **13f**.

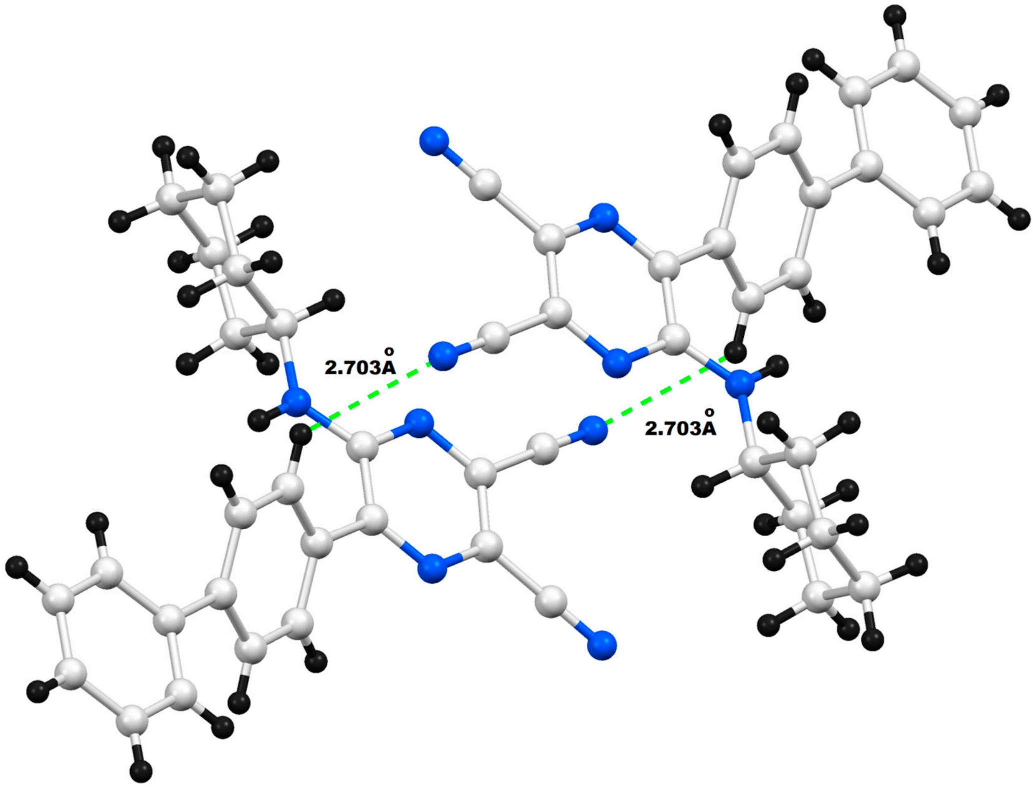

Figure S10. Intermolecular H-bonds experienced by **13f** molecules in its crystal network.

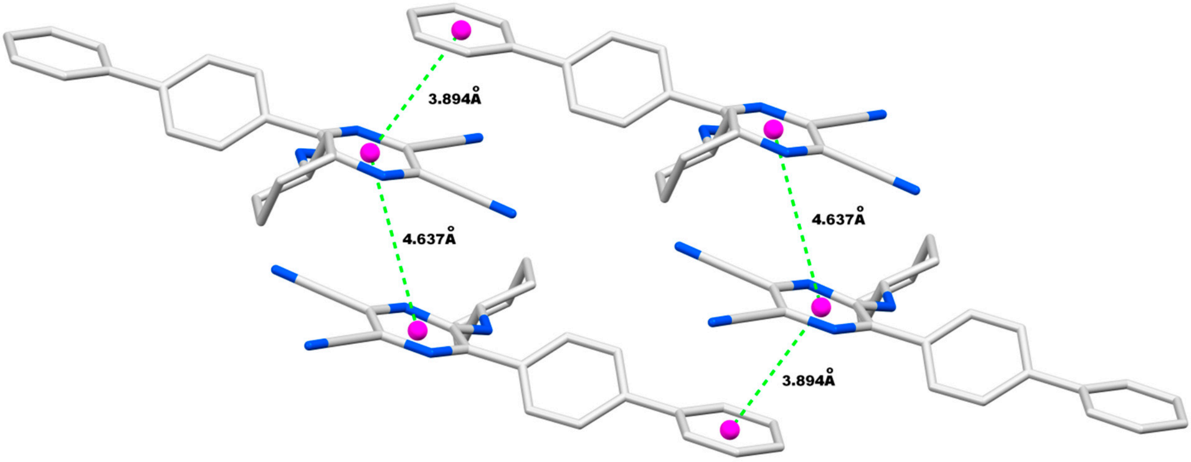

Figure Sl1. π - π interactions among **13f** molecules in their crystal network

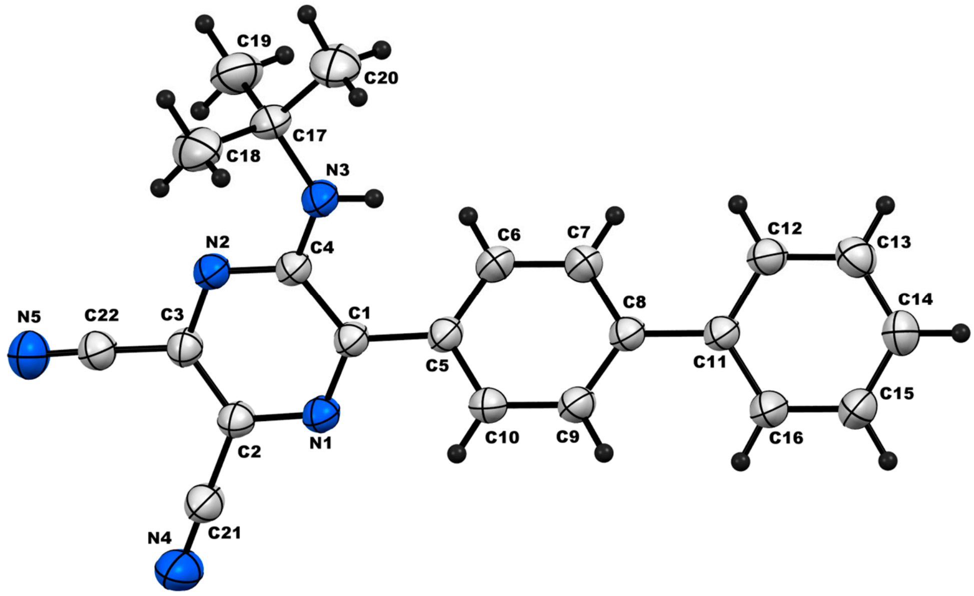

Figure S12. Crystal structure (Thermal ellipsoid representation) of **13k**.

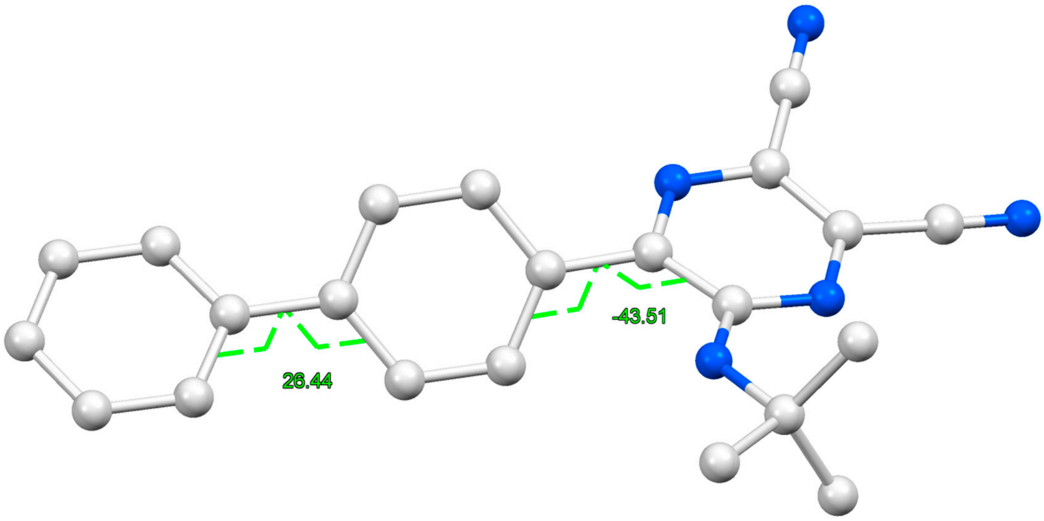

Figure S13. Orientation and the corresponding torsion angle of phenyl ring against the pyrazene ring along with the phenyl-phenyl orientation of the bi-phenyl moiety in the crystal structure of the molecule **13k**.

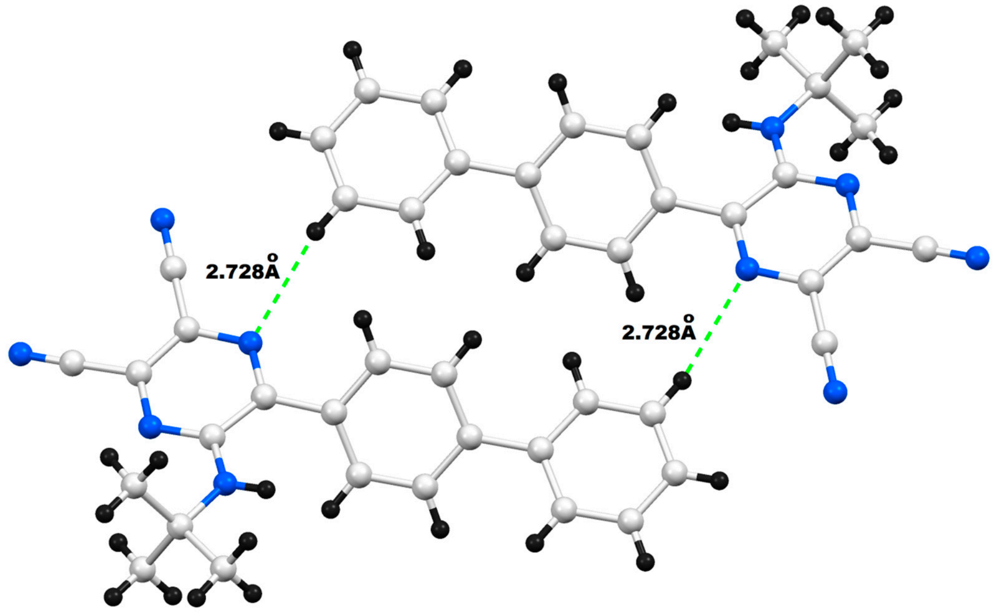

Figure S14. Intermolecular H-bonds experienced by **13k** molecules in its crystal network.

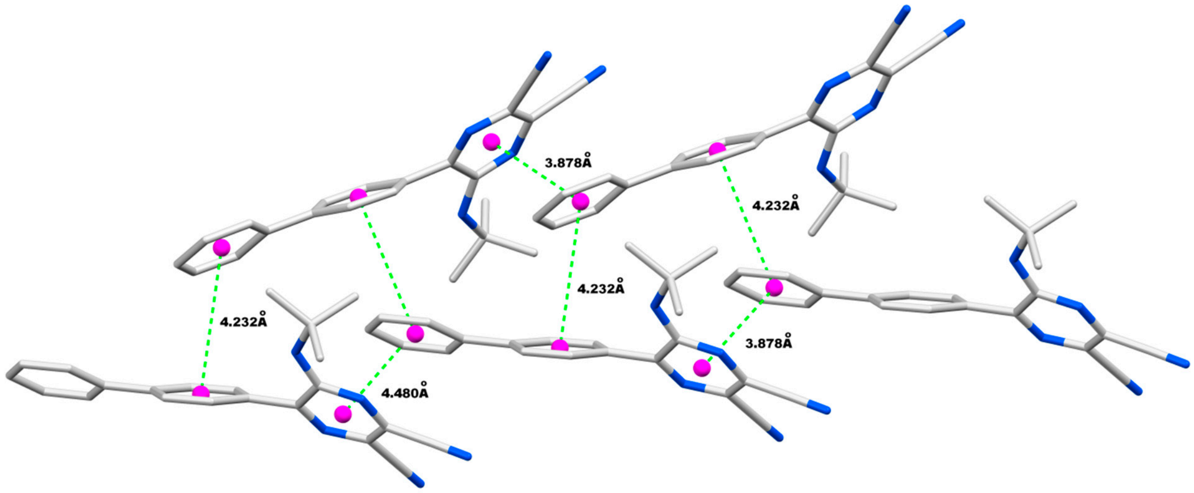

Figure S15. π - π interactions among **13k** molecules in their crystal network
